# Uncovering sequence effects in Titanium binding peptides adsorption on TiO_2_: A molecular dynamics study

**DOI:** 10.1038/s41598-025-10966-3

**Published:** 2025-07-24

**Authors:** Roja Rahmani, Alexander P. Lyubartsev

**Affiliations:** Department of Chemistry, Stockholm University, Svante Arrhenius väg 16C, Stockholm, 10691 Sweden

**Keywords:** Titanium dioxide, Peptides, Adsorption, Molecular dynamics, Computational chemistry, Molecular dynamics, Atomistic models, Organic-inorganic nanostructures

## Abstract

Titanium binding peptides are useful tools for material functionalization in both biomedical and nanotechnology applications because of their ability to attach selectively to titanium surfaces. In this work, we investigate the adsorption behavior of a series of 360 six amino acids long peptides obtained by permutations of titanium binding peptide residues, RKLPDA, on hydroxylated anatase $\hbox {TiO}_2$ (101) surfaces using extensive atomistic molecular dynamics (MD) simulations, with the purpose identifying sequences with stronger adsorption affinity to titanium. Our results show that small changes in amino acid order can significantly affect both binding strength and structural conformations. Peptides with arginine at the N-terminus and lysine or aspartic acid near the C-terminus tended to exhibit more stable adsorption. The clustering and radial distribution function (RDF) analyzes revealed different binding modes and key atomic interactions, with nitrogen-containing groups and, in some cases, $\hbox {Na}^{+}$ ions playing a significant role in the anchoring of peptides to the surface. These findings suggest a detailed sequence-level understanding of peptide-$\hbox {TiO}_{2}$ interactions and can guide the design of improved peptides for titanium functionalization.

## Introduction

Solid binding peptides (SBPs) are short amino acid sequences that can specifically recognize and bind to a wide range of solid surfaces. In general, SBPs bind to solid surfaces via multiple non-covalent interactions. In physiological environments, SBPs can enhance the biocompatibility of hybrid materials and be efficiently engineered to select and recognize specific interfaces. SBPs can be used to initiate and control the synthesis of composite materials or as molecular linkers to guide the simple and directed immobilization of biomolecules on solid surfaces^1–3^. These features make SBPs attractive for manufacturing bioinspired materials in diagnostic and therapeutic applications. SBPs have been extensively developed for a diverse range of applications in the areas of biotechnology, bio-sensing, biocatalysis, drug delivery, vaccine development, biomedical implants, bioimaging, and cell capture^4–6^.

Standard inert materials used in the production of orthopedic and dental implants include titanium (Ti) and titanium alloys. Due to titanium’s its excellent mechanical qualities, biocompatibility, and corrosion resistance, it is the perfect material for implantable devices. However, the absence of recognizable moieties on the implant prevents cell activities from being regulated and may induce a foreign body reaction to the implant, causing infections due to biofilm formation and bacterial adhesion. Achieving adhesion and selectivity at the interface of biomacromolecules and inorganic materials is vital to the construction of well-organized hybrid materials. The natural adhesion of peptides and proteins is the result of the evolutionary process. As such, these solid-binding peptides may not possess the required properties for biomedical applications.

Titanium-binding peptides have gained significant interest because of their potential applications in various fields, particularly biomedicine and material science. No specific binders have evolved for titanium in nature. Therefore, it is important to design novel peptides that specifically bind to titanium. There are several techniques to identify and tailor SBPs, such as isolating the functional domains of naturally occurring proteins, creating customized peptides with amino acid sequences that are not present in nature and have desired binding properties to the solid using biophysical and biological principles and computational methods (de novo design), and combinatorial phage display method, where a diverse set of random amino acid sequences are tested and a small number of candidates selected based on how well they bind to the target material. Other designed peptides are inspired by biomineralization, where the interaction of precursor ions interacts with biomacromolecules, which results in the growth of some inorganic layers. Moreover, throughput computational screening tools like Rosetta, alphafold, etc., are increasingly used to guide the design of peptides and proteins.

Many studies have shown the adsorption of different peptides on titanium^7^. An established titanium-binding peptide (TBP), RKLPDA (min TBP-1), was introduced by Sano and Shiba^8^. First, they discovered a dodecamer sequence, RKLPDAPGMHTW, using the phage display method. They realized that the first six residues (RKLPDA), or the so-called min TBP-1, play a central role in the binding of dodecamer to Ti, and, by isolating the hexamer RKLPDA, they found that it binds as strongly as dodecamer. Min TBP-1 is known for its strong affinity for titanium surfaces, which makes it useful to improve the properties and biocompatibility of titanium-based materials. One end of the TBP specifically recognizes the surface of titanium, while the other end can be used to link with various bioactive molecules. Watanabe et al., by inserting min TBP-1 into the silk fibroin (SF) protein, prepared a titanium functionalizing material (TiBP-SF), which improved bone formation activity in osteoblasts-like cells (bone synthesizing) when coated on the surface of Ti plates^9^. In another study, Yoshinari et al. evaluated the binding behavior of conjugated molecules consisting of min TBP-1 and antimicrobial peptides on titanium surfaces in order to investigate the bioactivity of Porphyromonas gingivalis bacteria involved in inflammatory gum and peri-implant diseases. Their results indicated that modifying the titanium surface improved the antimicrobial activity of the surface against P. gingivalis and is a promising method to reduce biofilm formation on Ti surfaces^10^. Over the past few decades, several peptide tags and chemical modifications have been created torotein immobilize protein molecules. They have been used to purify proteins and monitor enzymatic reactions on lab-on-a-chip devices^11–13^. The orientation of the immobilized polymerase enzyme on a solid-state device has a significant impact on its enzymatic activity^14^. By coupling min TBP-1 with DNA polymerase, Hirokazu et al. designed a min TBP-1-tagged DNA polymerase that could not only stably immobilize DNA polymerase on the $\hbox {TiO}_2$ surface but also maintain its enzymatic activity^15^.

﻿In general, SBPs﻿ bind to solid surfaces via multiple non-covalent interactions. The success of finding the most suitable peptide for the given task depends on understanding the interactions between amino acids and the target material, and the great challenge with effectively utilizing SBPs is in controlling the relationship between peptide sequences and their functionality. Various factors, including the surface topography of the solid and surrounding solution conditions, influence the binding strength of solid binding peptides. Sampath et al. highlighted the importance of the surface chemistry of $\hbox {TiO}_2$ on the binding strength of peptides and, in particular, the distribution of hydroxyl groups due to the dissociation of water molecules at the interface^16^. They calculated the binding free energies of the 6mer, min TBP-1 (RKLPDA), and 12mer (RKLPDAPGMHTW) on neutral hydroxylated (NH) ($\sim$ −10 and $\sim$ −12 kJ/mol) and negative non-hydroxylated (NeNH) (−23 and 21 kJ/mol) of rutile surfaces using molecular dynamics (MD) simulations. In agreement with prior experiments and simulations, they found that these two peptides bind with almost the same strength to the same surface. However, their binding free energies are significantly different when it comes to different surfaces of rutile with different charges. The peptides adsorbed weaker to the roxylatedsurface (NeNH) when they bound primarily through the arginine (R) and lysine (K) residues, whereas on the surface with higher affinity of peptides (NH), they bound primarily through the arginine and aspartate (D) amino acids. This aligns with previous experimental studies showing that the binding free energy is lower when the residues involved are arginine and lysine compared to binding with arginine and aspartate, which results in higher binding strengths. This study denoted the importance of having detailed information about the surface (such as charge, coverage with hydroxyl, etc.) of reactive materials such as titania. However, a pivotal determinant in the binding process is the composition of these peptides, encompassing the sequence of amino acids, structural conformation, and charge. Hayashi et al. elucidated the underlying mechanism of binding of ferritin molecules whose N-terminal domain is endowed with min TBP-1 and titanium surface as substrate. They showed that min TBP-1 maintains its specificity towards the titanium surface even after being introduced into the N-terminal domain of ferritin. Also, by conducting adhesion force analysis using atomic force microscopy (AFM), they showed that electrostatic interactions between R and D groups of min TBP-1 and the titanium surface, and they strongly bound to charges originating from the protonation and deprotonation of the surface groups of a Ti substrate, in agreement with the mechanism proposed by Sano and Shiba^17^. Mirau et al., using Nuclear Overhauser Effects (NOEs) NMR spectroscopy, determined the three-dimensional structure of the bound peptide at the surface of titania , which adopts a C-shaped conformation (in agreement with Sano and Shiba), realizing that lysine has little or no effect on this bound state^8,18^. By performing Saturation Transfer Difference (STD) NMR studies, Suzuki et al. characterized the interactions of a min TBP-1 with $\hbox {TiO}_2$ nanoparticles, which have a negative zeta potential on the surface. They found that the positively charged guanidyl group of arginine (R) and the amino group of lysine (K) play key roles in interaction with the $\hbox {TiO}_2$ surface due to electrostatic interactions between the negative surface and positive sidechains. However, they did not find the aspartic acid (D) residue binding to the surface significantly different from the binding mechanism that Sano and Shiba initially proposed^19^. Brandt et al. performed unbiased MD and Metadynamics simulations of amino acids’ side chains and min TBP-1 on the rutile (100) surface to investigate the collective effects of amino acids on peptide adsorption. They found out that the total adsorption free energy of the peptide is −11.3 kJ/mol, which is stronger than the individual contributions of side chain amino acids^20^. They have also mentioned that the compact binding mode is stabilized on the surface due to the salt bridge formation between ARG and ASP amino acids. Schneider et al. manifested that the reason behind the selectivity and binding preference of min TBP-1 in Ti over Si is primarily influenced by solvent-mediated interactions at the interfaces. Using molecular dynamics simulations, they revealed that the min TBP-1 peptide binds selectively to titanium over silicon due to variations in local solvent structure. Adsorption free energies and adhesion forces on rutile and silicon surfaces were quantified using Metadynamics and steered MD simulations, aligning well with experimental results. Unlike previous assumptions focusing on electrostatics, the study emphasized the peptide’s ability to sense the interfacial solvent structure at an atomic scale^21^. Polimeni et al. investigated the dynamics of the AMRKLPDAPGMHC peptide sequence at the anatase ($\hbox {TiO}_2$) 100 surface using unbiased MD simulations. The adsorption process begins with the peptide diffusing from the bulk water phase toward the $\hbox {TiO}_2$ surface, followed by its attachment to the surface . This attachment is facilitated by interfacial water layers, which interact with the charged side chain groups of the peptide^22^.

In this study, we address two major gaps in studies of the adsorption of titanium binding peptides on titanium surfaces. Although previous investigations have focused extensively on the adsorption behavior of the RKLPDA (minTBP-1) peptide on rutile surfaces, adsorption on anatase has been much less studied. This is significant given the structural and surface chemistry differences between rutile and anatase, as well as the fact that the anatase form is prevalent in small nano-sized $\hbox {TiO}_2$ nanoparticles. Furthermore, while the role of individual amino acids in the RKLPDA sequence has been explored using different experimental approaches and alanine substitution, the effect of sequence order on adsorption strength has not been systematically studied. Here, we explore 360 different permutations of the six amino acids in RKLPDA to identify those with stronger adsorption than the original sequence, RKLPDA. We first generated all 720 possible permutations of the six amino acids in RKLPDA (R, K, L, P, D, A). To avoid redundancy, we treated reverse sequences as equivalent in terms of composition. For example, RKLPDA and ADPLKR contain the same residues in opposite order. However, because the peptide backbone is not symmetric upon reversion and because the peptides in our simulations are modeled in zwitterionic form with charged N- and C-termini, reversed sequences are not structurally identical. Despite this distinction, we chose to include only one sequence from each reverse pair for computational feasibility. The selection was made arbitrarily during sequence building. This resulted in a final dataset of 360 unique, and directionally distinct sequences, with about uniform distribution of amino acids over the six positions in the peptide. Although many studies of adsorption involved biased simulations such as umbrella sampling or metadynamics to compute the potential of mean force and adsorption free energies, in this work we utilized unbiased atomistic MD. Unbiased simulations have the advantages of more straightforward evaluation of canonical averages such as radial distribution functions, statistics of different events, kinetic properties, and their comparison across different systems. Through this approach, we aim to provide a comprehensive understanding of sequence-dependent adsorption behavior and to identify peptides with enhanced affinity for $\hbox {TiO}_2$ anatase surfaces.

## Results and discussions

### Surface separation distance and end-to-end distance analysis

**Fig. 1:**
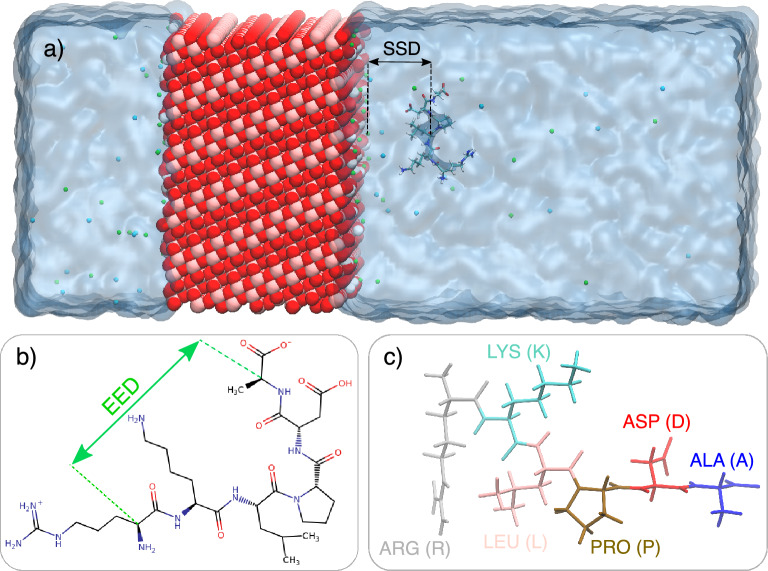
Components of the simulated systems for RKLPDA (min TBP-1) peptide. (**a**) simulation box; slab of anatase 101 hydroxylated surface, water shown as ice blue area), RKLPDA peptide, $\hbox {Na}^{+}$ and $\hbox {Cl}^{-}$ ions represented as green and blue spheres. (**b**) sketch of the RKLPDA peptide (**c**) respresentation of composing amino acids of RKLPDA peptide.

We first analyze the behavior of the peptides near the $\hbox {TiO}_2$ surface in terms of the distance between the center of mass of the peptides and the $\hbox {TiO}_2$ surface (Surface Separation Distance, SSD), and End-to-End Distances (EED) of the peptides. The $\hbox {TiO}_2$ surface was determined as the layer of the outmost Ti atoms of the slab. EED was calculated as the distance the between $\hbox {C}_\alpha$ atoms of the first and last amino acids in the sequence, as shown in Fig. 1.

**Fig. 2:**
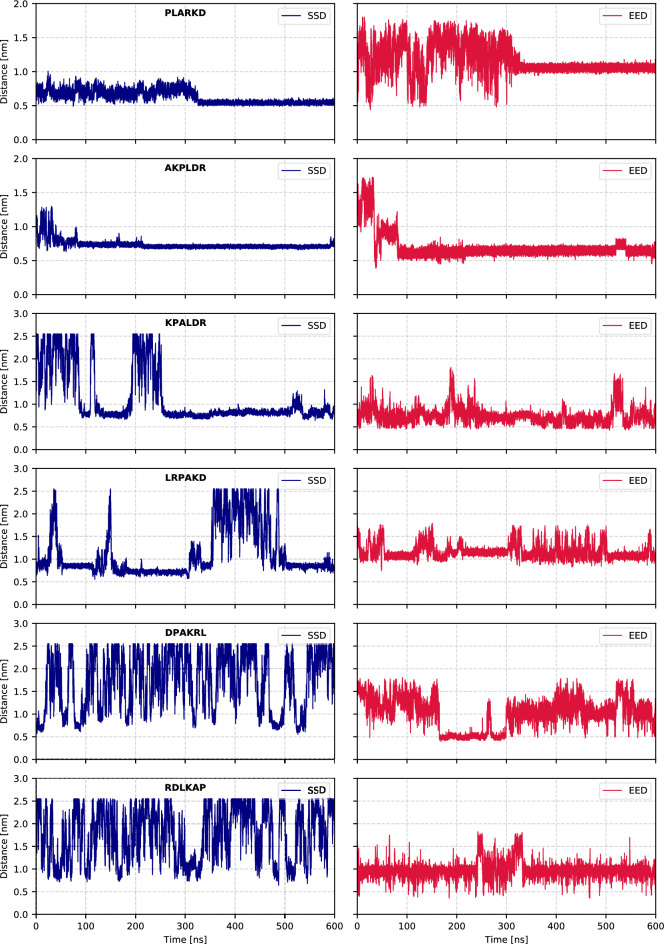
Examples of SSD and EED as function of the simulation time for permanently bound (PLARKD and AKPLDR), repeatedly binding and unbinding (KPALDR and LRPAKD), and weakly binding (DPAKRL and RDLKAP) peptides.

An example of the time evolution of SSD and EED for some selected peptides is shown in Fig. 2.

We conventionally determined the adsorbed (bound) state of the peptide if SSD is less than 1 nm. When the peptide is not bound to the surface, it changes SSD between close (within 1 nm) and far (about 2.5 nm) states on a nanosecond time scale, repeatedly approaching the surface during 600 ns simulation. We observed that, depending on the sequence, some peptides already become bound to the surface after first contact, other peptides adsorb and desorb several times, and some peptides do not stay near the surface any longer. We have ranked the binding potency of the peptides by determining the percentage of time when they stay bound to the surface with SSD below 1 nm during the 600 ns trajectory. The complete ranking list of all 360 peptides according to this criterion is given in Table S1 of the Supporting Information. The maximum value of approximately ∼2.5 nm in the SSD plots corresponds to half the simulation box length in the z-direction (excluding the slab thickness). It is worth mentioning that even when peptides travel to the opposite side of the box, they effectively interact with the other top surface of the slab, which is identical.

Figure S1 of the Supporting Information represents the SSD and EED data for all 360 peptides, along with the percentage of simulation time during which the SSD remains below 1 nm during the 600 ns trajectory. The analysis reveals that 138 out of 360 sequences spent more than 50% of the simulation time close to $\hbox {TiO}_2$.

To gain insight into how adsorption affects the conformation of peptides, we analyzed their end-to-end distance separately for bound and unbound states. Table 1 presents the average EED values of peptides adsorbed to the surface (SSD < 1 nm, denoted $\hbox {EED}_{a}$) and desorbed (SSD $\ge$ 1 nm, $\hbox {EED}_{d}$), as well as standard deviations of EEDs for 50 top-ranking peptides according to their adsorption potency. The comparison of $\hbox {EED}_{a}$ and $\hbox {EED}_{d}$ reveals several interesting trends. In many sequences, $\hbox {EED}_{a}$ is noticeably lower than $\hbox {EED}_{d}$, indicating that peptides tend to adopt more compact conformations when adsorbed. This compaction likely reflects structural rearrangement upon surface binding, where residues come closer together to maximize interaction with the surface, either through side-chain contacts or terminal interactions. For example, PAKRLD and RPLAKD both show low EED values in the bound state consistent with the compact structures observed in cluster analysis ($\sim$0.5–0.65 nm, see below). In contrast, peptides such as ADKPLR and KALPDR maintain higher EED values during adsorption ($\sim$1.4 nm), reflecting more extended or linear conformations even when in close proximity to the surface. These differences likely stem from sequence-specific properties: for instance, proline is known to introduce rigidity in the peptide backbone because of its cyclic structure, which restricts rotation and can prevent compact folding. Likewise, the location of charged residues (such as ARG, LYS, or ASP) can influence electrostatic interactions and stabilize certain conformations over others. Interestingly, a few sequences show little change in EED between bound and unbound states (e.g., RPKADL or KPLDAR), suggesting that some peptides retain similar structural characteristics regardless of their proximity to the surface. This suggests that some peptides may have intrinsically rigid structures that naturally favor surface binding without requiring major conformational changes. Overall, our results highlight that adsorption-induced structural changes are strongly influenced by the peptide sequence. Measurement of end-to-end distance provides a straightforward yet informative way to capture these conformational differences. Peptides that adopt more compact structures upon adsorption may interact more effectively with the surface by allowing multiple residues or termini to make contact simultaneously with the suitable fragment of the surface structure. These insights can support the rational design of peptides with enhanced surface-binding properties for material functionalization and related applications.

**Table 1:** Peptides binding ranking according to the percentage of time to have SSD value less than 1 nm over production part of the simulation (% bound) together with average EED in adsorption ( $\hbox {EED}_{a}$) and desorption ( $\hbox {EED}_{d}$) region. The standard deviation of EED values in adsorption $\hbox {std}_{a}$ and desorption $\hbox {std}_{d}$ region are shown as well.

Sequence	% bound	$\hbox {EED}_{a}$	$\hbox {std}_{a}$	$\hbox {EED}_{d}$	$\hbox {std}_{d}$	Sequence	% bound	$\hbox {EED}_{a}$	$\hbox {std}_{a}$	$\hbox {EED}_{d}$	$\hbox {std}_{d}$
PLARKD	100.00	1.16	0.22	1.15	-	RPKLDA	87.53	0.74	0.16	0.74	0.20
RLDKAP	99.56	0.98	0.21	1.30	0.25	PDKRLA	87.53	1.21	0.25	1.39	0.15
DPKLRA	99.14	0.94	0.17	1.49	0.15	KRALDP	87.47	1.37	0.10	1.09	0.22
PKRALD	98.36	1.15	0.11	1.32	0.18	KLRPDA	87.30	1.15	0.19	1.26	0.19
RPLDKA	98.09	0.79	0.20	1.20	0.21	APDKLR	87.26	0.87	0.21	1.07	0.23
PRLKAD	97.90	1.31	0.26	1.46	0.13	DPALKR	87.04	0.92	0.18	1.21	0.23
KRPLAD	97.76	1.05	0.07	1.23	0.17	RKALDP	86.32	0.96	0.36	1.14	0.32
AKPLDR	97.52	0.68	0.15	1.46	0.13	RKAPLD	86.03	1.00	0.16	1.34	0.21
RLAPKD	96.76	1.17	0.21	1.46	0.10	KPDLAR	85.32	0.69	0.11	0.73	0.15
ADKPLR	96.70	1.39	0.17	1.52	0.15	RPALDK	83.82	0.78	0.11	0.76	0.22
RKLPDA	96.51	1.17	0.16	1.48	0.14	LPAKRD	83.22	1.21	0.19	1.24	0.24
LPDARK	96.41	0.84	0.23	1.34	0.16	AKLPRD	83.08	1.19	0.19	1.43	0.19
RADPKL	96.10	1.26	0.22	1.52	0.10	LKRDAP	83.05	1.56	0.10	1.32	0.17
KPRLDA	95.93	1.04	0.26	1.33	0.15	PKLDRA	81.84	0.80	0.32	1.24	0.32
PAKRLD	95.63	0.84	0.39	1.35	0.20	KLPADR	81.09	1.12	0.22	1.31	0.21
RPLAKD	95.55	0.66	0.10	0.92	0.27	LDRPKA	80.73	1.30	0.21	1.46	0.13
RADPLK	94.34	1.30	0.22	1.43	0.14	RPLKDA	80.72	0.75	0.21	0.75	0.19
RPDLAK	93.99	0.76	0.13	0.82	0.15	KDRAPL	80.71	1.21	0.25	1.49	0.13
RLAKPD	93.02	0.96	0.17	1.16	0.30	LAKRDP	80.69	0.94	0.19	1.37	0.26
DAKRPL	92.74	1.15	0.17	1.40	0.17	KARPDL	80.49	1.31	0.19	1.38	0.15
RAKPLD	92.64	1.22	0.19	1.46	0.10	KLDRPA	80.47	1.39	0.20	1.20	0.22
RKAPDL	91.96	1.31	0.27	1.46	0.14	APRDKL	79.95	0.79	0.23	1.09	0.32
KALPDR	91.73	1.33	0.19	1.44	0.13	DRKALP	79.90	1.26	0.23	1.30	0.29
PDKRAL	91.40	1.24	0.27	1.31	0.22	ADRLKP	79.79	1.01	0.24	1.19	0.23
RADKPL	88.43	1.11	0.26	1.36	0.19	AKDPLR	78.40	1.11	0.30	1.24	0.31

### Effect of aminoacids sequence on the binding potency

We first analyze the effect of the positioning of each amino acid in the peptide sequence. From the data in Table 1 one can observe that of the 25 peptides showing the highest binding ratio, 13 peptides have arginine (R) as the first amino acid, which in most cases is followed by alanine (A) or proline (P). The last peptide in the sequence for the strongest binding peptides is most often aspartic acid (D). However, among the 25 least binding peptides, there are only two peptides with arginine in the first position and 2 with aspartic acid in the last position. One can suggest that arginine in the first position and the N-terminal group, create the best conditions for binding to the $\hbox {TiO}_2$ surface.

**Fig. 3:**
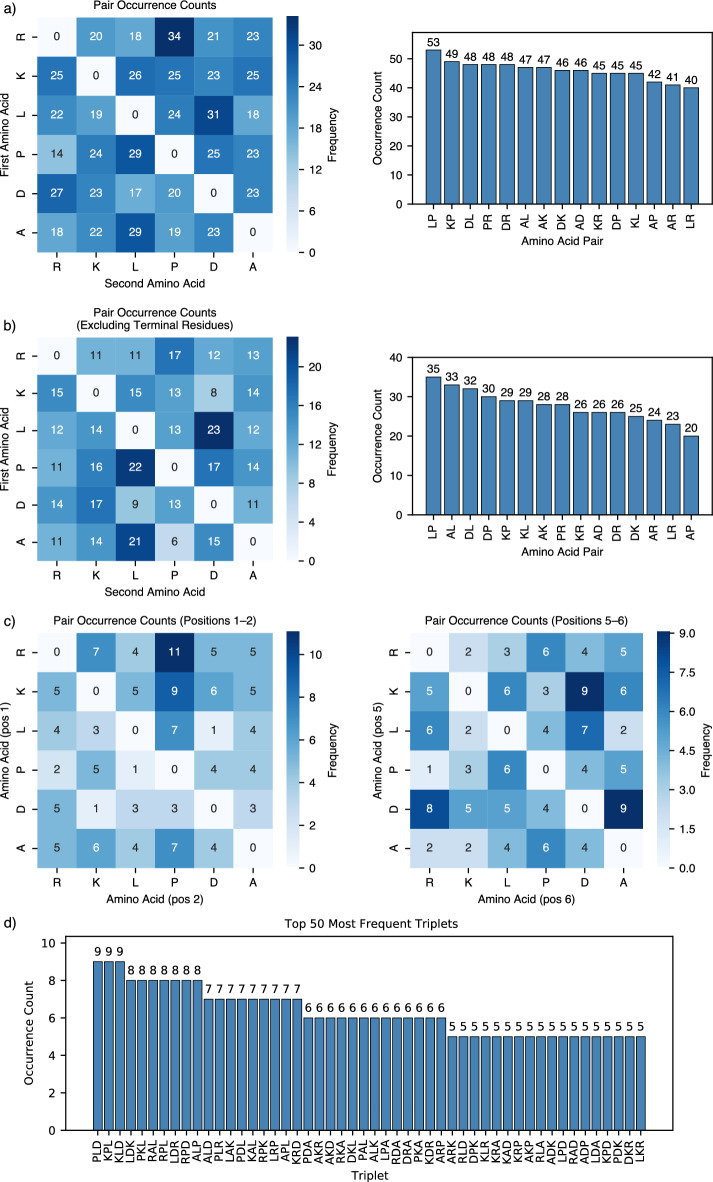
Pair occurrence counts for amino acids in peptides bound to the surface more than 50% of time: (**a**) across all positions (**b**) excluding terminal residues (**c**) at positions 1 and 2 (left) and 5 and 6 (right), (**d**) top 50 most frequent triplets.

**Fig. 4:**
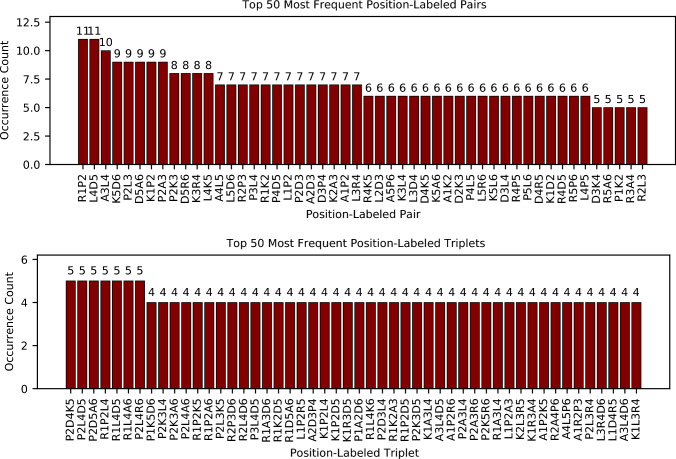
The 50 most common amino acid pairs (top) and triplets (bottom) are labeled by their positions within the sequence, for peptides bound to the surface more than 50% of time.

We have looked further at the occurrence of different subsequences of peptides that show stronger binding to $\hbox {TiO}_2$. Here, to obtain better statistics, we analyzed peptides that have SSD within 1 nm more than 50% of the observed time. Fig. 3 illustrates the frequency of pairs and triplets of amino acids in the sequences of these peptides. Fig. 3a presents the most common pairs of amino acids, considering both their sequential order (left) and total occurrence (right) but ignoring their position within the sequence. The pairs identified the most frequently are LP, KP, DL, PR, and DR. In contrast, Fig. 3b and Fig. 3c differentiate between sequence regions. Fig. 3b focuses on the middle section of sequences between positions 2 and 5, while Fig. 3c highlights the most frequent amino acids at the N-terminal (positions 1 and 2) and C-terminal (positions 5 and 6). The analysis reveals that sequences commonly start with $\hbox {R}_1$ $\hbox {P}_2$ and end with either $\hbox {K}_5$ $\hbox {D}_6$ or $\hbox {D}_5$ $\hbox {A}_6$. Additionally, Fig. 4 displays the most frequent positional pairs and triplets within the strongly adsorbed sequences.

### Clustering analysis

**Fig. 5:**
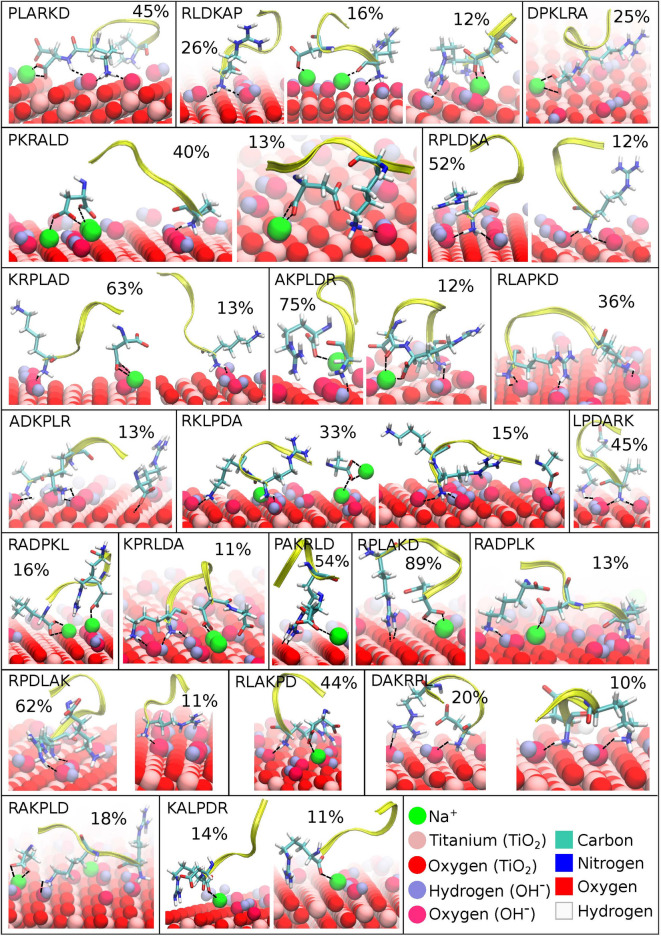
Bound-state conformations for the sequences with SSD < 1 nm for more than 90% of the simulation time (first 24 sequences in Table 2) obtained from clustering analysis. Only the most populated clusters (more than 10%) for each sequence is shown together with their populations. The $\hbox {TiO}_{2}$ surface atoms–including titanium, oxygen, and the oxygen and hydrogen atoms of surface hydroxyl groups–as well as sodium ions ($\hbox {Na}^+$), are depicted in ball representation and marked with circle shapes, following a consistent color scheme. The peptide backbones are shown in yellow, while only the amino acid residues in direct contact with the surface are displayed in cylinder representation, color-coded and indicated with square shapes. Dashed lines denote possible interactions, such as hydrogen bonding or coordination, between peptide atoms and surface atoms.

Cluster analysis has been performed in order to investigate whether peptides adopt a single dominant binding conformation or multiple binding modes. Fig. 5 and Fig. S2 of the Supporting Information show the representative bound-state conformations from different clusters for sequences that remained adsorbed (SSD < 1 nm) for over 90% of the simulation time. The corresponding SSD and EED values for each cluster are summarized in Table 2. Some sequences, such as PLARKD, DPKLRA, RLAPKD, ADKPLR, LPDARK, RADPKL, KPRLDA, PAKRLD, RPLAKD, RADPLK, RLAKPD and RAKPLD, were found to bind in a single conformation. Others, such as RLDKAP, PKRLAD, RPLDKA, KRPLAD, AKPLDR, RKLPDA, RPDLAK, DAKRPL, and KALPDR, exhibited two or even three distinct binding conformations, each with populations above 10%. These peptides displayed a range of structural preferences, from compact conformations such as PAKRLD to more extended ones like KALPDR. The structures in Fig. 5 reveal how diverse peptide-surface interactions can be. Some peptides adopt tightly packed, looped conformations that likely maximize favorable contacts with the surface, while others bind in more stretched-out forms. This suggests that there is no single preferred binding motif; instead, different sequences have their own best way of interacting with the surface, depending on their flexibility and residue composition. In several cases, such as RKLPDA, RLDKAP, and KRPLAD, we observed multiple distinct binding conformations, each representing a stable adsorption mode. This indicates that some sequences are conformationally adaptable, possibly shifting their orientation or anchoring residues in response to local electrostatics or hydration effects at the surface. Sodium ions ($\hbox {Na}^+$) also play a noticeable role in these interactions. In many structures, $\hbox {Na}^+$ acts as a bridge between negatively charged groups, like the carboxylate side chain of ASP or the C-terminus and the surface for some of peptides. For example, AKPLDR adsorbs via ARG, with coordination by $\hbox {Na}^+$, while in RKLPDA (min TBP-1), one cluster shows adsorption through ARG and LYS, with ALA involvement mediated by $\hbox {Na}^+$. These observations align well with previous studies by Shiba et al., who emphasized electrostatic interactions between ARG and hydroxyl groups, as well as ASP anchoring via its carboxylate^19^. Our RKLPDA conformations also closely resemble those reported by Sampath et al. on hydroxylated $\hbox {TiO}_2$ surfaces^16^, though ALA was not involved in their work due to terminal capping. Another factor influencing binding is the zwitterionic form of the peptides used in our simulations. Because the terminals are not capped, both N- and C-terminal groups are available for interaction. For instance, in RPDLAK and DAKRPL, LYS at the C-terminus anchors the peptide to the surface through interactions with negatively charged surface oxygens or hydroxyls. Side chains with oxygen atoms, such as ASP, can directly coordinate with titanium, while basic residues like ARG may bind through their nitrogen atoms. $\hbox {Na}^+$ ions further facilitate these interactions by bridging between the peptide and the surface. Moreover, due to the zwitterionic nature of the peptides, surface binding can occur regardless of the intrinsic polarity of individual side chains. For instance, although leucine (LEU) is non-polar and hydrophobic, when located at the terminal position–as in RADPKL–it can still interact electrostatically with the surface via its carboxyl group and a coordinating $\hbox {Na}^+$ ion. Similarly, in DAKRPL, although ASP typically binds through its side-chain carboxylate, when present at the N-terminus, it adsorbs via its amino group instead. Finally, there seems to be a connection between structural compactness and stronger or more stable binding. Peptides with tighter, more folded conformations often correspond to higher-population clusters, indicating that these compact structures might allow for enhanced cooperative interaction with the surface that through several binding groups that match the structure of the surface, which in turn stabilize the compact peptide structure.

**Table 2:** Clusters populations together with their corresponding SSD and EED for the sequences with SSD < 1 nm for more than 90% of the simulation time (first 24 sequences in Table 1) obtained from clustering. Only the most populated clusters (more than 10%) for each sequence are shown.

**Sequences**	**Cluster**	**Cluster Population(%)**	**SSD (nm)**	**EED (nm)**	**time (ns)**
PLARKD	Cluster 1	44.75	0.54	1.06	584505
	Cluster 1	26.28	0.80	1.01	29375
RLDKAP	Cluster 2	15.56	0.70	0.73	586165
	Cluster 3	12.30	0.69	1.25	392490
DPKLRA	Cluster 1	25.12	0.80	0.90	320860
PKRALD	Cluster 1	39.79	0.69	1.17	371580
	Cluster 2	12.70	0.68	1.14	437050
RPLDKA	Cluster 1	51.95	0.84	0.68	466035
	Cluster 2	12.22	0.89	0.96	486065
PRLKAD	-	-	-	-	-
KRPLAD	Cluster 1	63.37	0.77	1.05	181940
	Cluster 2	12.60	0.84	1.07	265030
AKPLDR	Cluster 1	75.23	0.69	0.65	463870
	Cluster 2	11.78	0.73	0.59	121370
RLAPKD	Cluster 1	36.49	0.65	1.02	369450
ADKPLR	Cluster 1	12.66	0.55	1.43	39610
RKLPDA	Cluster 1	32.61	0.65	1.05	100460
	Cluster 2	14.92	0.61	1.33	475740
LPDARK	Cluster 1	45.20	0.79	0.73	348135
RADPKL	Cluster 1	15.51	0.71	1.24	399515
KPRLDA	Cluster 1	10.76	0.72	0.93	358450
PAKRLD	Cluster 1	53.91	0.84	0.50	368775
RPLAKD	Cluster 1	89.10	0.77	0.65	433175
RADPLK	Cluster 1	13.21	0.73	0.69	533940
RPDLAK	Cluster 1	61.87	0.73	0.74	392785
	Cluster 2	11.25	0.70	0.74	98135
RLAKPD	Cluster 1	44.31	0.74	0.83	284395
DAKRPL	Cluster 1	19.85	0.79	1.09	459725
	Cluster 2	10.06	0.63	1.17	141345
RAKPLD	Cluster 1	17.89	0.65	1.38	367355
RKAPDL	-	-	-	-	-
KALPDR	Cluster 1	14.35	0.85	1.51	563730
	Cluster 2	11.08	0.73	1.23	377925
PDKRAL	-	-	-	-	-

### Radial distribution functions

**Fig. 6:**
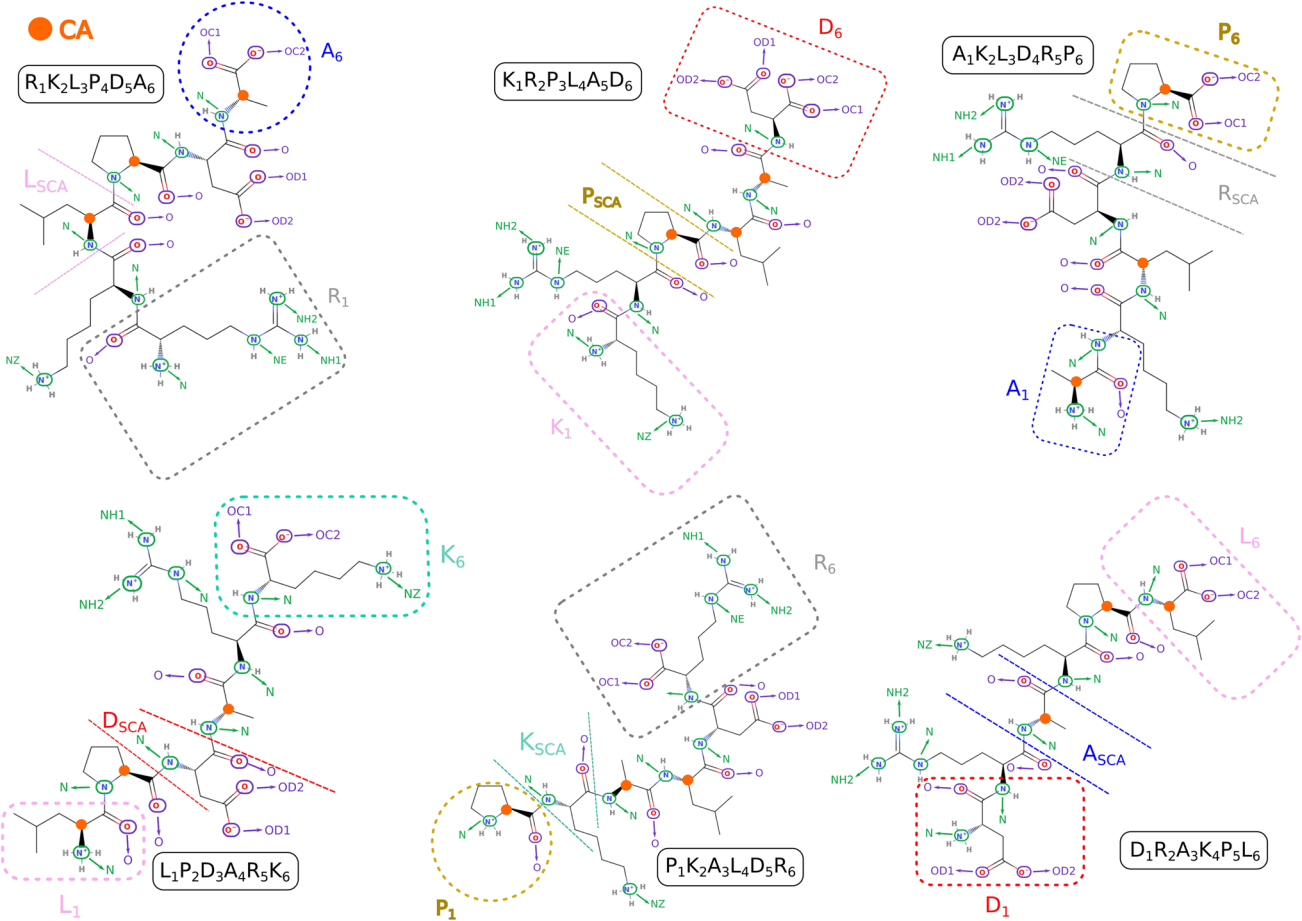
Notation guide for RDF of different atoms in amino acids. Notations for atoms of amino acids at different positions including N-terminal, middle (SCA), and C-terminal are shown.

**Fig. 7:**
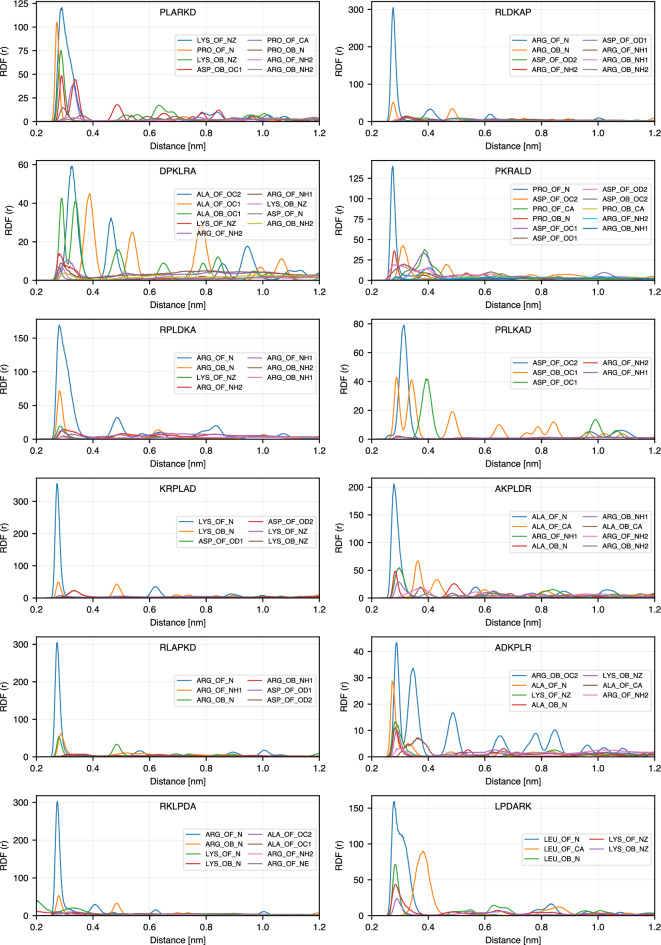

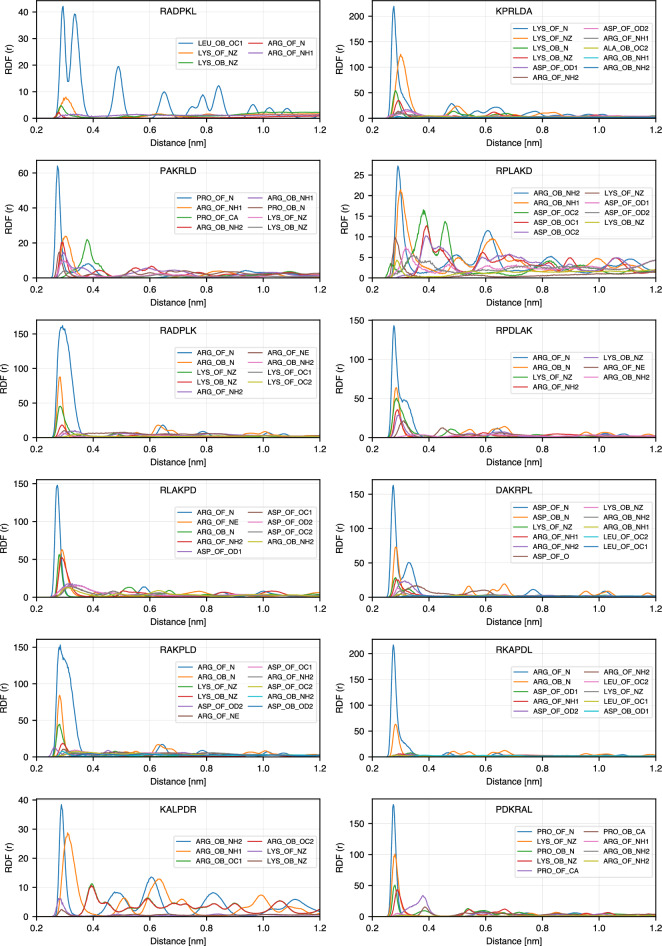
RDFs of the top 24 sequences in the Table 1.

To get further insight into the atomistic details of the peptide binding, we calculated radial distribution functions (RDF) between selected atoms of the peptides: N atoms of basic amino acids (ARG and LYS) and amino acids at the N-terminus, oxygen atoms of the acidic amino acid (ASP) and amino acids at C-terminus, $\hbox {C}_{\alpha }$ for non-charged amino acids (ALA, LEU and PRO), and oxygen atoms of $\hbox {TiO}_2$ surface: bridge oxygens (OB) and oxygens of the hydroxyl groups (OF). On the anatase-101 surface, oxygen atoms are more exposed to the environment than Ti atoms and provide favorable possibilities for binding of polar peptide atoms through hydrogen bonds. Fig. 6 explains the notations used for RDFs between different atoms in the peptide sequences and $\hbox {TiO}_2$ surface oxygens. The list of RDFs with the highest RDF maximum for each peptide is provided in Tables S1 and S2 of the Supporting Information, and RDFs for 24 highest ranking binding peptides are shown in Fig. 7. In this figure, only RDFs with the maxima occurring at distances less than 4 Å are shown. The complete RDF dataset is available as stated in the Data Availability section.

Our analysis shows that the highest RDF peaks are observed for atoms binding to the oxygen of hydroxyl groups on the surface (atom type OF), while lower (but still high) maximum RDF values are often observed for bridging oxygens (atom type OB). This indicates that adsorption is driven mostly by the hydroxyl groups at the surface, and thus is expected to be dependent on pH. This conclusion is in agreement with the experimental findings of pH-dependent amino acids adsorption on the $\hbox {TiO}_2$ surface^23^, and in particular with the fact that Lysine adsorption increases with pH when the amount of OH groups is expected to increase.

Among the peptides atoms that show the highest RDF peaks, more than 85% are nitrogen atoms, either from the N-terminus or the side chains of amino acids. This can also be seen in Fig. 5, where in most sequences the basic N-containing amino acids (ARG and LYS) are anchored or close to the surface, together with the amino acids at the N-terminus (ALA in ADKPLR, PRO in PKRALD and PLARKD, LEU in LPDARK, etc.). N-terminus atoms show systematically high RDF peaks even for less strongly bound peptides. Furthermore, examining other high peaks in each sequence reveals that other atoms in amino acids also exhibit first RDF maxima close to the surface. As shown in Fig. 7, each of the strongly bound peptides has multiple amino acids that contribute to surface adsorption. This observation is consistent with the bound-state conformations presented in Fig. 5. For example, PLARKD displays high RDF peaks within 0.4 nm for four amino acids, including K, P, D, and R, corresponding to their proximity to the surface in the bound state (see Fig. 5). Similarly, in RKLPDA, two amino acids, R and D, have peaks within 0.4 nm, which is consistent with the peptide conformation in the bound state, Fig. 5. One can note that, although the A residue also regularly appears close to the surface, it is anchored to the surface via $\hbox {Na}^{+}$ resulting in a slightly larger distance from the surface oxygen atoms OB and OF. This is further analyzed in the next Section.

### Effect of $\hbox {Na}^+$ on adsorption

**Fig. 8:**
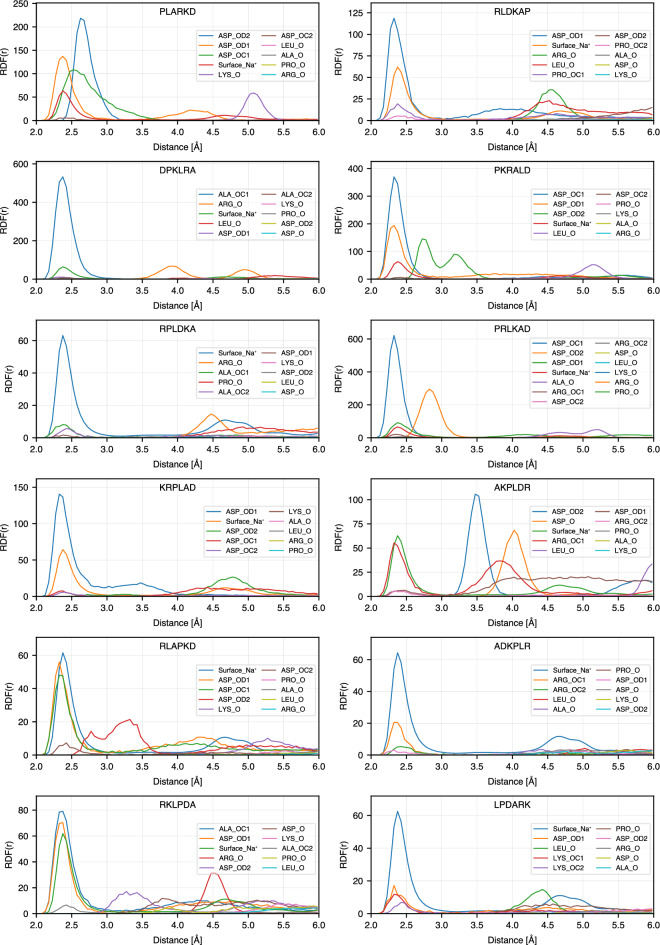

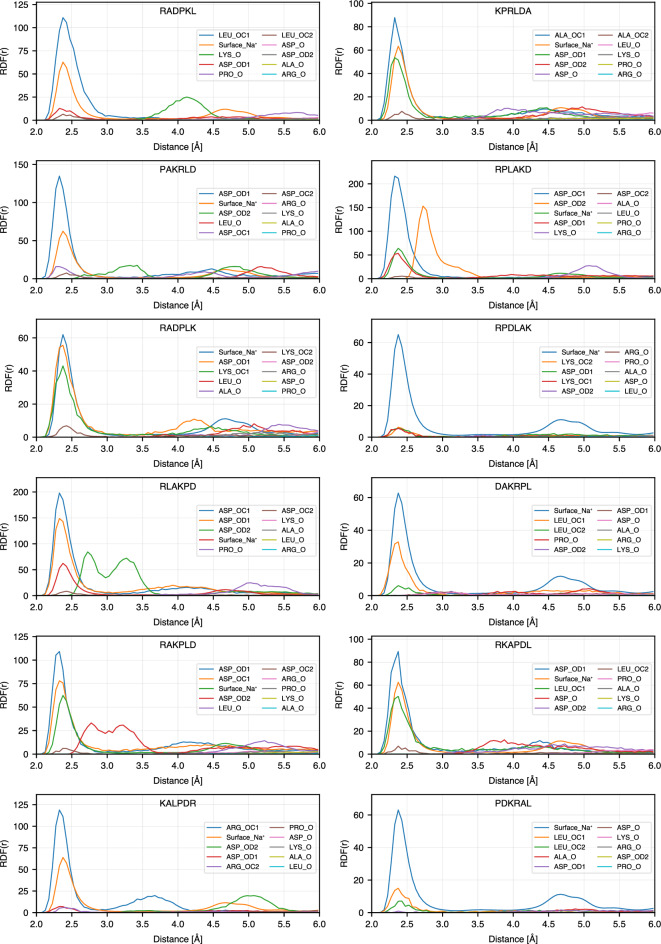
RDFs of oxygen atoms in 24 top-ranking sequences and surface $\hbox {Na}^+$.

To investigate the role of $\hbox {Na}^{+}$ ions in the adsorption of peptides on the $\hbox {TiO}_{2}$ surface, we analyzed conditional radial distribution functions between sodium ions on the surface ($\hbox {Na}^{+}_{\text {surface}}$) and oxygen atoms of peptides and $\hbox {TiO}_2$ surface. For these calculations, $\hbox {Na}^{+}$ ions were filtered based on a dual distance criterion: only ions located within 3 Å of either of the surface oxygen atoms (OB and OF), and simultaneously within 3 Å of any peptide oxygen (e.g., backbone or sidechain oxygens), were selected for the analysis. Fig. 8 shows these RDFs calculated for 24 peptides that remained adsorbed to the surface (SSD < 1 nm) for more than 90% of the simulation time. Compared with the RDFs in Fig. 8 with the RDFs in Fig. 7 and the bound-state conformations shown in Fig. 5, we observe that in some sequences, $\hbox {Na}^{+}$ ions appear to play a significant role in mediating peptide adsorption. For example, the sequences DPKLRA, PKRALD, and PRLKAD show strong RDF peaks for peptide oxygen atoms (especially from ASP side chains or C-terminal groups) that overlap in position and intensity with those of $\hbox {Na}^{+}$. This spatial overlap and comparable peak intensity suggest that these peptides are likely stabilized through $\hbox {Na}^{+}$ bridging interactions with the surface. In contrast, other peptides, such as RPLDKA, RLAPKD, ADKPLR, LPDARK, RPDLAK, and DAKRPL, show much weaker or negligible RDF peaks for peptide oxygen atoms near $\hbox {Na}^{+}_{\text {surface}}$. This implies that $\hbox {Na}^{+}$ is not directly involved in mediating adsorption for the dominant bound-state conformation of these sequences. These observations are consistent with the conformations in Fig. 5, where sodium ions are absent or distant from the key adsorption sites. Furthermore, the specific types of oxygen atoms that contribute to the RDF peaks vary between sequences. In most cases, the high peak of $\hbox {Na}^{+}$ - peptide oxygen RDF is seen for ASP oxygen atoms, which are known to interact strongly with cations. However, there are notable exceptions. For example, in DPKLRA and KPRLDA, the dominant contribution comes from the C-terminal carboxyl oxygen in ALA, while in RADPKL this contribution comes from LEU. These results indicate that, although ASP is a primary anchor point, other residues, particularly at the terminal positions, can also participate in $\hbox {Na}^{+}_{\text {surface}}$-mediated adsorption, depending on their spatial proximity and accessibility. In general, the comparison between Fig. 8 and Fig. 5 highlights the sequence-specific nature of $\hbox {Na}^{+}$-facilitated adsorption. Some peptides rely on sodium ions to stabilize their interaction with the surface, while others bind directly via side chains or terminal groups without significant ionic mediation. This distinction is reflected not only in the RDF profiles but also in the clustering and structural conformations observed in the bound states. Together, these findings highlight the importance of considering both direct and ion-mediated contacts when characterizing peptide-surface interactions at the atomic level.

The observed sequence-dependent binding behavior can be understood from the chemical interactions between the peptides and the hydroxylated $\hbox {TiO}_2$ surface. Positively charged residues such as arginine (R) exhibits strong surface affinity, particularly when located at the N-terminus, due to the ability to form electrostatic interactions and multiple hydrogen bonds with the surface hydroxyl groups through the side-chain NH groups as it can be seen in the representative snapshots in Figure 5. As noted in the RDF plots (Figures 7), the oxygen atoms of surface hydroxyl groups (OF) play a significant role in facilitating these interactions. Arginine’s guanidinium group, in particular, contributes to stable binding by anchoring the peptide, as seen in sequences such as RLDKAP, PAKRLD, and RPLAKD. Lysine, with its positively charged side chain, can also form hydrogen bonds with surface OH groups, as observed in KPRLDA, RKLPDA, and related sequences. Another important factor is the presence of the free amino group at the N-terminus, which is positively charged at physiological pH and can directly interact with the surface so as the carboxyl groups at C-terminus which bind through sodium ions. This is evident in sequences such as DAKRPL, PKRLAD, and PLARKD, which, despite not starting with R or K side chains, still high in the ranking due to contributions from the terminal amine group. The negatively charged residue aspartic acid (D) also participates in surface binding, typically through sodium ion ($\hbox {Na}^+$)-mediated interactions. This can be seen in sequences such as RAKPLD and PAKRLD, as well as in others like RKLPDA and AKPLDR, which do not possess D at C-terminus but still exhibit binding via their C-terminal carboxyl groups. Due to the zwitterionic nature of the peptides, the terminal carboxyl group can coordinate with $\hbox {Na}^{+}$ ions and contribute to surface adsorption. This bridging mechanism is evident in the RDF profiles (Fig. 8), where $\hbox {Na}^+$ ions accumulate near carboxylate oxygen atoms, and is consistent with the binding conformations illustrated in Fig. 5. Overall, these findings highlight the key roles of surface chemistry, charge complementarity, and specific residue–side chain interactions in governing peptide adsorption.

## Conclusions

In this study, we systematically investigated the adsorption behavior of 360 possible permutations of the titanium-binding peptide RKLPDA on the surfaces of hydroxylated anatase $\hbox {TiO}_{2}$ (101) using extensive atomistic molecular dynamics simulations. Our findings highlight the strong sequence dependence of peptide adsorption, with 138 peptides showing more than 50% binding over the simulation time and several sequences exhibiting enhanced binding compared to the original RKLPDA. We identified sequence motifs and residue positions, particularly arginine at the N-terminus and lysine or aspartic acid at the C-terminus, that contribute to stronger and more stable adsorption. Clustering analysis revealed that high-binding peptides often adopt compact conformations to provide favorable contact with the surface, while RDF analysis confirmed the importance of basic nitrogen-containing residues (ARG, LYS) and the N-terminus of peptides in binding to $\hbox {TiO}_2$. We further found that hydroxyl groups on the surface facilitate binding of peptides by nitrogen-containing groups, while $\hbox {Na}^{+}$ ions were found to facilitate adsorption in a sequence-specific manner, bridging between negatively charged side chains and the $\hbox {TiO}_{2}$ surface in several peptides. Additionally, the zwitterionic form of peptides resulted in the adsorbance through amino acids that are intrisically non-polar and more hydrophobic so that the presence of amino group at N-terminus and carboxyl groups at C-terminus improved the adsorption tendency to the surface.

We should further note that the length of our simulations, 600 ns for each peptide, can provide reliable distinguishing between strongly and weakly binding peptides and a qualitative ranking of their binding potency; however, it is likely not enough to quantitatively characterize the strength of binding of strongly binding peptides in terms of binding free energies. Such computations need to be carried out by biased simulations such as Metadynamics, and we are planning to carry out such computations for strongly bound peptides identified in this study in the follow-up work.

Furthermore, the large amount of data obtained in this work, including non-biased molecular dynamics trajectories, instant interaction energies, RDFs, other structural and dynamical information obtained for each amino acid sequence, can contribute to subsequent data-driven analysis to develop machine learning models which would greatly facilitate investigation of various aspects of peptide-$\hbox {TiO}_2$ interactions.

Together, our work provides detailed insights into the molecular mechanisms of peptide-surface interactions and offers a computational framework for optimizing solid-binding peptide sequences for surface functionalization applications. These insights can be extended to the design of next-generation biomaterials with improved specificity and binding affinity.

## Materials and methods

### Titania surface

In this work, we investigate the binding of peptides on a fully hydrated and partially hydroxylated $\hbox {TiO}_2$ anatase (101) surface. The (101) anatase surface has the lowest surface energy and prevails in typical anatase nanoparticles^24^. Furthermore, Grote et al. reported that the density profile of water around the $\hbox {TiO}_2$ nanoparticle is the most similar to the previously obtained density profile for anatase (101) plane surface, both computed by *ab initio* molecular dynamics simulations^25,26^.

The $\hbox {TiO}_2$ atoms were arranged in a two-dimensional periodic slab with (101) surface exposed to water. We set the fraction of hydroxyl groups on the surface of anatase (101) as 30% to match the experimentally observed surface charge of $\hbox {TiO}_2$ nanoparticle at neutral pH of −0.62 e/$\hbox {nm}^2$^27^. The hydroxyl groups were randomly attached to 5-coordinated Ti surface atoms. Each surface has dimensions of approximately 4.92 $\times$ 4.94 $\times$ 3.05 $\hbox {nm}^3$ including 6 layers of Ti atoms. In this work, the force field parameterized by Rouse et al.^28^ based on previously performed ab initio molecular dynamics (DFT) simulations of several $\hbox {TiO}_2$ surfaces in water^26^ was used.

### Peptides

We performed MD simulations of 360 six amino acids long peptides $\hbox {X}_1$ $\hbox {X}_2$ $\hbox {X}_3$ $\hbox {X}_4$ $\hbox {X}_5$ $\hbox {X}_6$, where $\hbox {X}_i$={R, K, L, P, D, A} and R is arginine (ARG), K lysine (LYS), L leucine (LEU), P proline (PRO), D aspartic acid (ASP) and A is alanine (ALA,) obtained by permutations of amino acids present in titanium binding peptide RKLPDA. The peptides were in zwitterionic form, so the two ends of the peptides known as the N-terminus and C-terminus are not capped and are charged to mimic physiological conditions at pH=7.5 and 37 $^{\circ }$C. At neutral pH, ARG (pKa = 12.4) and LYS (pKa = 10.5) carry a positive charge, and ASP (pK=3.7) has a negative charge. The amino acids and peptides were built using Pymol^29^ and simulated using the Amber03 force field^30^. Lorentz-Berthelot combining rules were used for cross-interactions.

### The system

Each simulated system consisted of anatase $\hbox {TiO}_2$ slab, one of peptides and water, which was presented by the TIP3P model. Furthermore, $\hbox {Na}^{+}$ and $\hbox {Cl}^{-}$ ions were added to neutralize the system and provide a physiologically relevant ion concentration of 0.15 M. The simulation systems composed in this way, as well as their components, are shown in Fig. 1. Each of the systems consisted of $\sim$ 25000 atoms. An example of system compositions is provided in Table 3. For visualizing the systems, the Visual Molecular Dynamics (VMD) package has been used^31^.

**Table 3:** Size and composition of the simulated system (an example).

Box size ($\hbox {nm}^3$)	5.11$\times$4.95$\times$10.70
Slab thickness (nm)	3.05
$\hbox {TiO}_2$	2340 Ti, 4680 O
$\hbox {OH}^-$ (bound to Ti-surface)	78
$\hbox {H}_2$O	6106
$\hbox {Na}^+$	57
$\hbox {Cl}^-$	26
Peptide	1

### Simulations

All molecular dynamics simulations were performed using Gromacs simulation package, version 2022^32^. The SSD and EED distances were extracted from the simulation trajectories using the PLUMED v.2.7 plugin^33^. The parameters of all MD simulations were the following. A time step was set to 2 fs. The LINCS algorithm was used to constrain the bonds to hydrogen atoms^34^. Electrostatic interactions were computed using the Particle Mesh Ewald (PME) algorithm with a real-space cutoff of 1.0 nm and a grid resolution of 0.12 nm. The same cutoff was used to calculate short-range Lennard-Jones interactions^35^. After the initial configuration of all components, the steepest descent algorithm was used for energy minimization to remove unfavorable contacts. This was followed by an equilibration run at 300 K and 1 bar using the Berendsen thermostat and barostat^36^ for 3 ns. The last frame of that simulation was used for the subsequent NVT with the Nosé–Hoover thermostat, which lasted 600 ns^37,38^.

### Clustering algorithm

We performed clustering analysis to investigate the distinct conformations that contribute to the adsorbed state of the peptides. The clustering process was carried out as follows. Initially, frames corresponding to the adsorbed state, that is, when the SSD is less than 1 nm were extracted. Clustering was performed using the gmx cluster tool in GROMACS with an RMSD cutoff of 0.15 nm applied to the entire peptide structure. The Gromos clustering algorithm was employed, which iteratively identifies the clusters. The algorithm selects the first cluster as the structure with the largest number of neighbors within the cutoff distance. This structure and its neighbors are then excluded from subsequent iterations, and the process is repeated until all frames are assigned to certain clusters^39^. This analysis enabled us to quantify the structural diversity of the adsorbed conformations.

## Supplementary Information

Supplementary Information.

## Data Availability

All relevant data, including files to start and run simulations, initial and final configurations, data files for SSD and EED as function of time, RDFs, Jupyther notebooks, are provided in an archive deposited at Zenodo repository, https://doi.org/10.5281/zenodo.15267234
