# Supplementary material for: Uncovering sequence effects in Titanium binding peptides adsorption on TiO2: A molecular dynamics study
Source: Sci Rep. 2025 Jul 24;15:26885. doi: 10.1038/s41598-025-10966-3 (PMC12290007; doi:10.1038/s41598-025-10966-3)
Supplement: Supplementary file 1 — Supplementary Information. [file 41598_2025_10966_MOESM1_ESM.pdf]

Supporting Information for: Uncovering Sequence Effects in Titanium Binding peptides adsorption on TiO_2: A Molecular Dynamics Study

Roja Rahmani[1] and Alexander P. Lyubartsev[2*]

[1,2]Department of Chemistry, Stockholm University, Svante Arrhenius väg 16C, Stockholm, 10691, Sweden.

*Corresponding author(s). E-mail(s): alexander.lyubartsev@su.se;
Contributing authors: roja.rahmani@su.se;

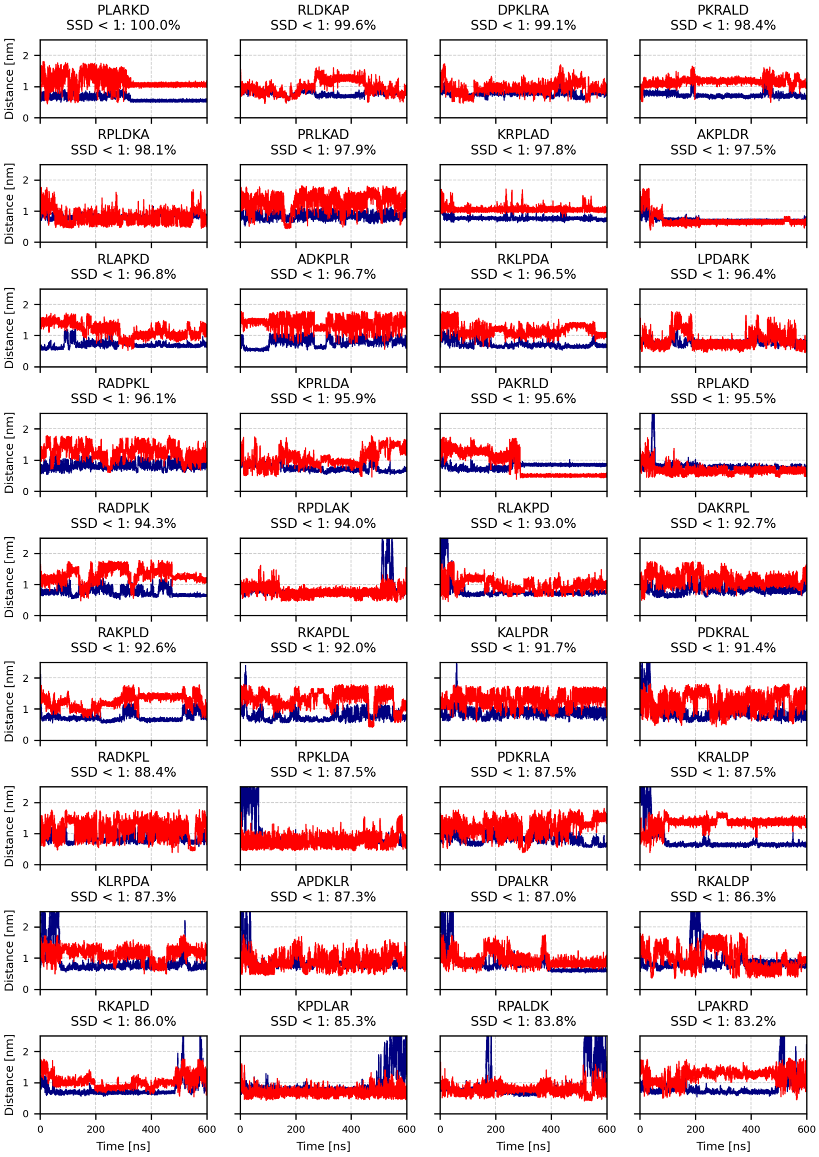
PLARKD
SSD < 1: 100.0%
RLDKAP
SSD < 1: 99.6%
DPKLRA
SSD < 1: 99.1%
PKRALD
SSD < 1: 98.4%
RPLDKA
SSD < 1: 98.1%
PRLKAD
SSD < 1: 97.9%
KRPLAD
SSD < 1: 97.8%
AKPLDR
SSD < 1: 97.5%
RLAPKD
SSD < 1: 96.8%
ADKPLR
SSD < 1: 96.7%
RKLPDA
SSD < 1: 96.5%
LPDARK
SSD < 1: 96.4%
RADPKL
SSD < 1: 96.1%
KPRLDA
SSD < 1: 95.9%
PAKRLD
SSD < 1: 95.6%
RPLAKD
SSD < 1: 95.5%
RADPLK
SSD < 1: 94.3%
RPDLAK
SSD < 1: 94.0%
RLAKPD
SSD < 1: 93.0%
DAKRPL
SSD < 1: 92.7%
RAKPLD
SSD < 1: 92.6%
RKAPDL
SSD < 1: 92.0%
KALPDR
SSD < 1: 91.7%
PDKRAL
SSD < 1: 91.4%
RADKPL
SSD < 1: 88.4%
RPKLDA
SSD < 1: 87.5%
PDKRLA
SSD < 1: 87.5%
KRALDP
SSD < 1: 87.5%
KLRPDA
SSD < 1: 87.3%
APDKLR
SSD < 1: 87.3%
DPALKR
SSD < 1: 87.0%
RKALDP
SSD < 1: 86.3%
RKAPLD
SSD < 1: 86.0%
KPDLAR
SSD < 1: 85.3%
RPALDK
SSD < 1: 83.8%
LPAKRD
SSD < 1: 83.2%
Distance [nm]
Time [ns]
0
1
2
0
200
400
600

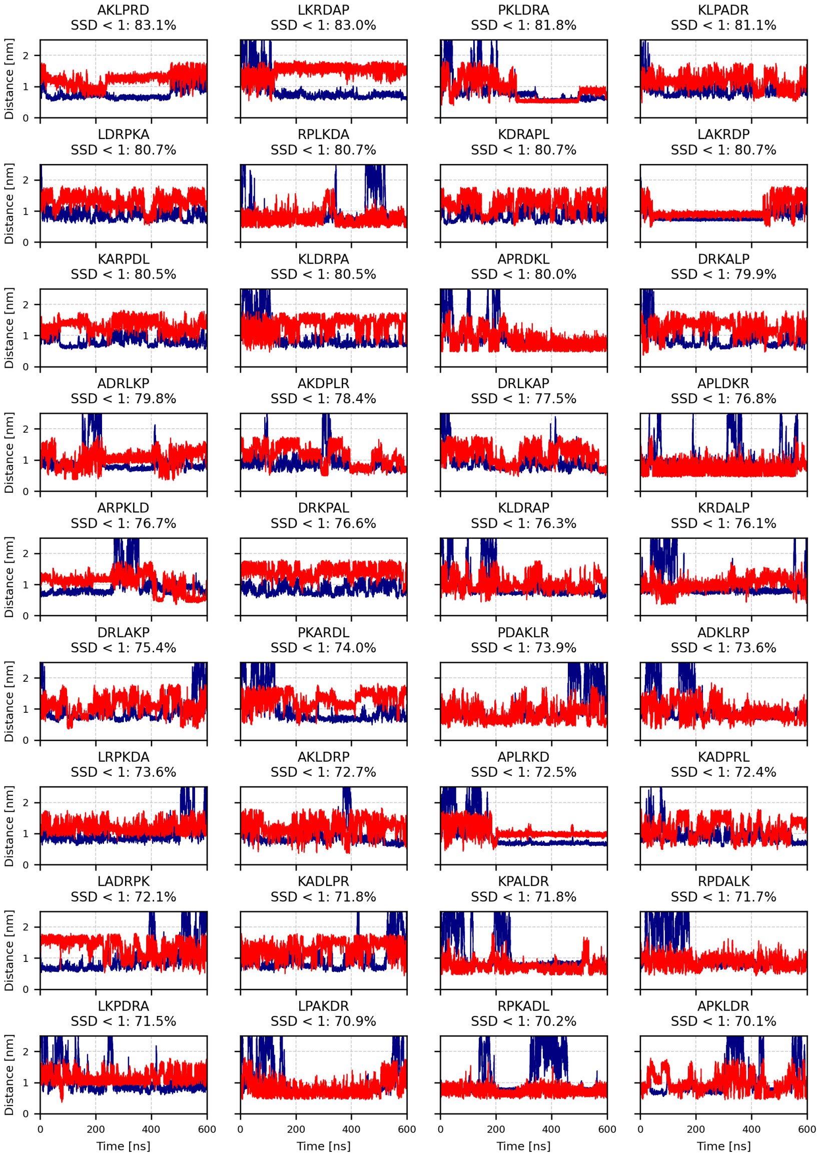
AKLPRD
SSD < 1: 83.1%
LKRDAP
SSD < 1: 83.0%
PKLDRA
SSD < 1: 81.8%
KLPADR
SSD < 1: 81.1%
LDRPKA
SSD < 1: 80.7%
RPLKDA
SSD < 1: 80.7%
KDRAPL
SSD < 1: 80.7%
LAKRDP
SSD < 1: 80.7%
KARPDL
SSD < 1: 80.5%
KLDRPA
SSD < 1: 80.5%
APRDKL
SSD < 1: 80.0%
DRKALP
SSD < 1: 79.9%
ADRLKP
SSD < 1: 79.8%
AKDPLR
SSD < 1: 78.4%
DRLKAP
SSD < 1: 77.5%
APLDKR
SSD < 1: 76.8%
ARPKLD
SSD < 1: 76.7%
DRKPAL
SSD < 1: 76.6%
KLDRAP
SSD < 1: 76.3%
KRDALP
SSD < 1: 76.1%
DRLAKP
SSD < 1: 75.4%
PKARDL
SSD < 1: 74.0%
PDAKLR
SSD < 1: 73.9%
ADKLRP
SSD < 1: 73.6%
LRPKDA
SSD < 1: 73.6%
AKLDRP
SSD < 1: 72.7%
APLRKD
SSD < 1: 72.5%
KADPRL
SSD < 1: 72.4%
LADRPK
SSD < 1: 72.1%
KADLPR
SSD < 1: 71.8%
KPALDR
SSD < 1: 71.8%
RPDALK
SSD < 1: 71.7%
LKPDRA
SSD < 1: 71.5%
LPAKDR
SSD < 1: 70.9%
RPKADL
SSD < 1: 70.2%
APKLDR
SSD < 1: 70.1%
Distance [nm]
Time [ns]
0
1
2
200
400
600

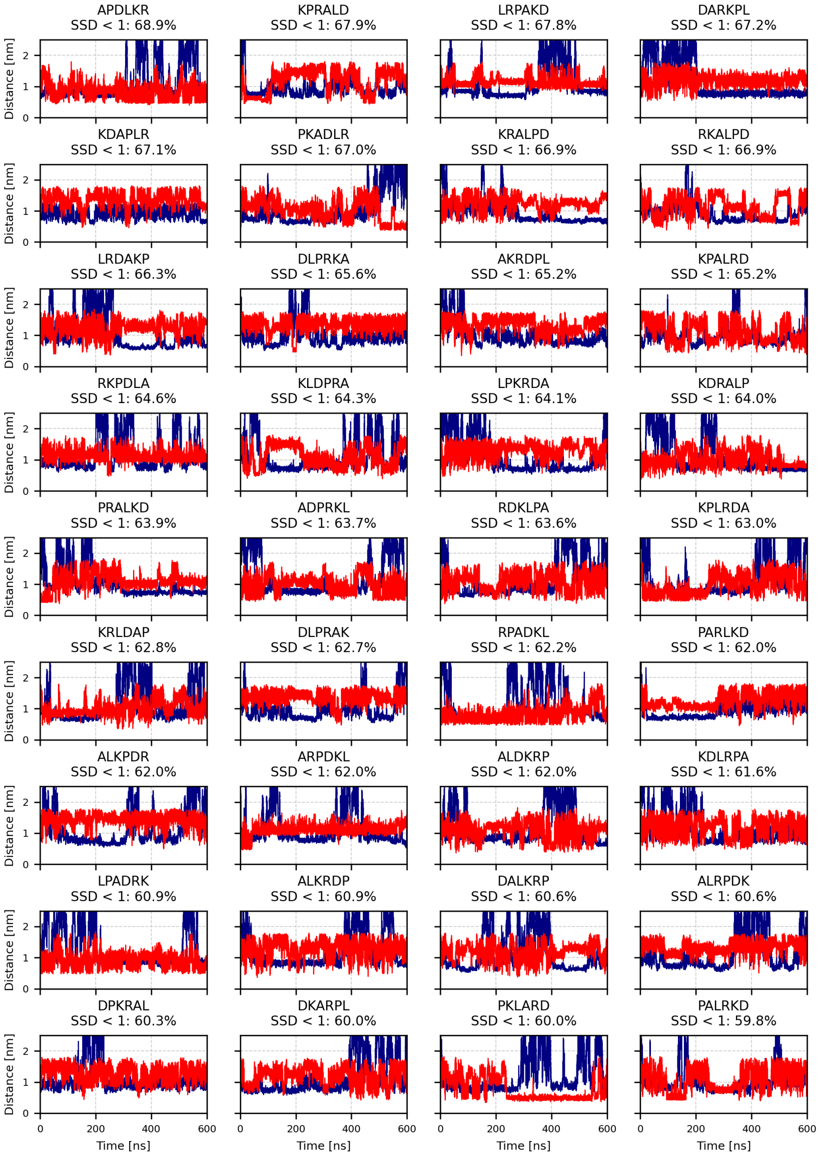
APDLKR SSD < 1: 68.9%
KPRALD SSD < 1: 67.9%
LRPAKD SSD < 1: 67.8%
DARKPL SSD < 1: 67.2%
KDAPLR SSD < 1: 67.1%
PKADLR SSD < 1: 67.0%
KRALPD SSD < 1: 66.9%
RKALPD SSD < 1: 66.9%
LRDAKP SSD < 1: 66.3%
DLPRKA SSD < 1: 65.6%
AKRDPL SSD < 1: 65.2%
KPALRD SSD < 1: 65.2%
RKPDLA SSD < 1: 64.6%
KLDPRA SSD < 1: 64.3%
LPKRDA SSD < 1: 64.1%
KDRALP SSD < 1: 64.0%
PRALKD SSD < 1: 63.9%
ADPRKL SSD < 1: 63.7%
RDKLPA SSD < 1: 63.6%
KPLRDA SSD < 1: 63.0%
KRLDAP SSD < 1: 62.8%
DLPRAK SSD < 1: 62.7%
RPADKL SSD < 1: 62.2%
PARLKD SSD < 1: 62.0%
ALKPDR SSD < 1: 62.0%
ARPDKL SSD < 1: 62.0%
ALDKRP SSD < 1: 62.0%
KDLRPA SSD < 1: 61.6%
LPADRK SSD < 1: 60.9%
ALKRDP SSD < 1: 60.9%
DALKRP SSD < 1: 60.6%
ALRPDK SSD < 1: 60.6%
DPKRAL SSD < 1: 60.3%
DKARPL SSD < 1: 60.0%
PKLARD SSD < 1: 60.0%
PALRKD SSD < 1: 59.8%
Distance [nm]
Time [ns]

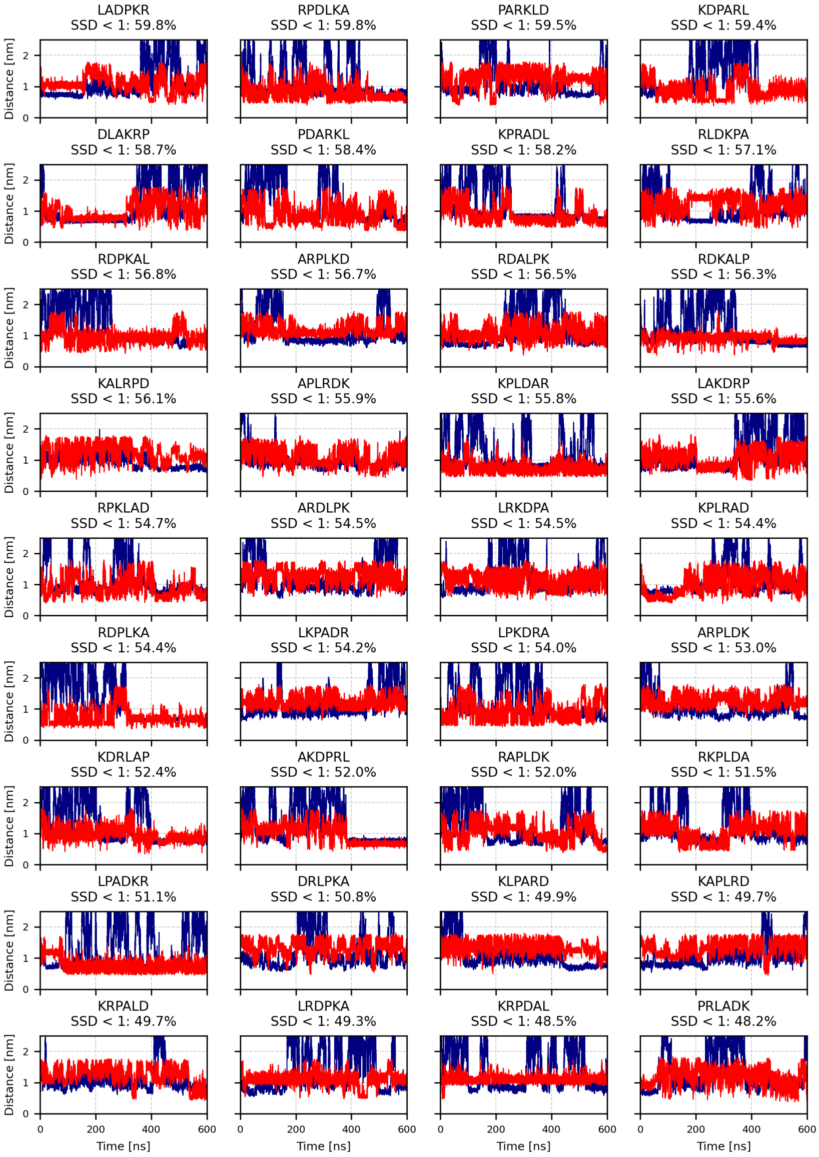
LADPKR
SSD < 1: 59.8%
RPDLKA
SSD < 1: 59.8%
PARKLD
SSD < 1: 59.5%
KDPARL
SSD < 1: 59.4%
DLAKRP
SSD < 1: 58.7%
PDARKL
SSD < 1: 58.4%
KPRADL
SSD < 1: 58.2%
RLDKPA
SSD < 1: 57.1%
RDPKAL
SSD < 1: 56.8%
ARPLKD
SSD < 1: 56.7%
RDALPK
SSD < 1: 56.5%
RDKALP
SSD < 1: 56.3%
KALRPD
SSD < 1: 56.1%
APLRDK
SSD < 1: 55.9%
KPLDAR
SSD < 1: 55.8%
LAKDRP
SSD < 1: 55.6%
RPKLAD
SSD < 1: 54.7%
ARDLPK
SSD < 1: 54.5%
LRKDPA
SSD < 1: 54.5%
KPLRAD
SSD < 1: 54.4%
RDPLKA
SSD < 1: 54.4%
LKPADR
SSD < 1: 54.2%
LPKDRA
SSD < 1: 54.0%
ARPLDK
SSD < 1: 53.0%
KDRLAP
SSD < 1: 52.4%
AKDPRL
SSD < 1: 52.0%
RAPLDK
SSD < 1: 52.0%
RKPLDA
SSD < 1: 51.5%
LPADKR
SSD < 1: 51.1%
DRLPKA
SSD < 1: 50.8%
KLPARD
SSD < 1: 49.9%
KAPLRD
SSD < 1: 49.7%
KRPALD
SSD < 1: 49.7%
LRDPKA
SSD < 1: 49.3%
KRPDAL
SSD < 1: 48.5%
PRLADK
SSD < 1: 48.2%
Distance [nm]
0
1
2
Time [ns]
0
200
400
600

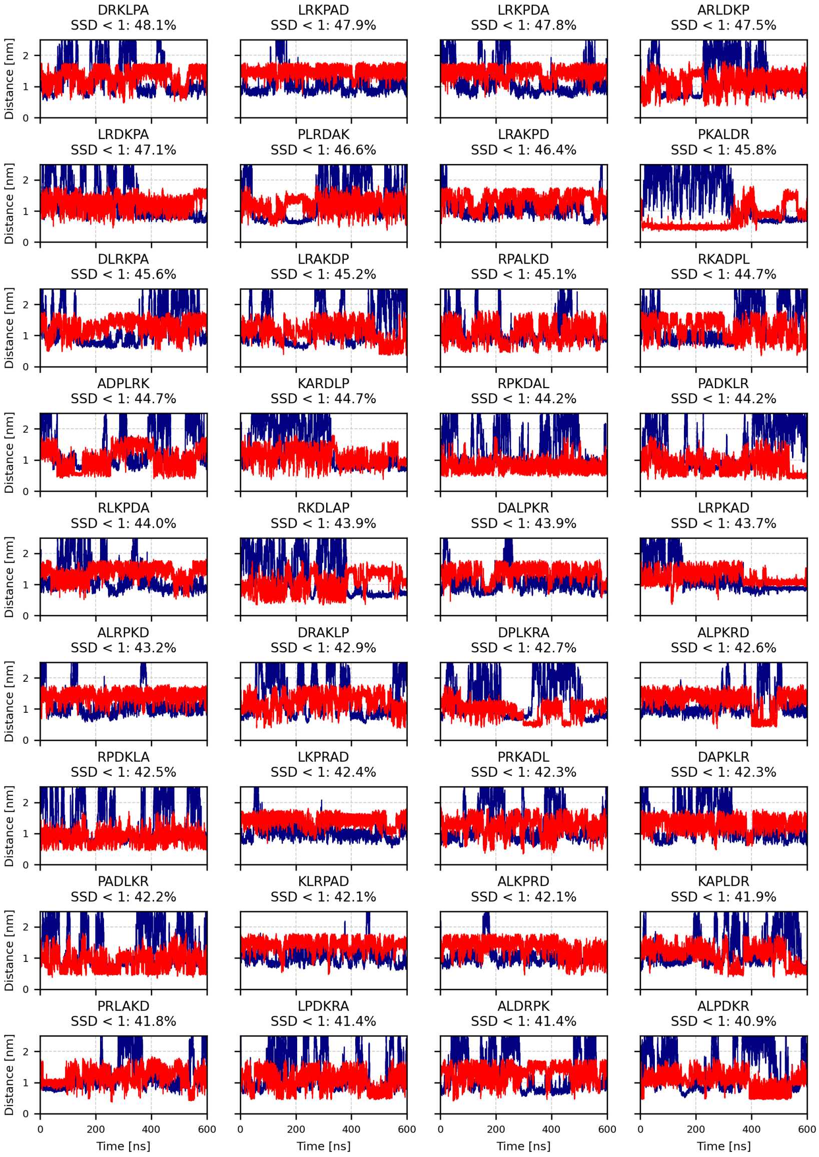
DRKLPA
SSD < 1: 48.1%
LRKPAD
SSD < 1: 47.9%
LRKPDA
SSD < 1: 47.8%
ARLDKP
SSD < 1: 47.5%
LRDKPA
SSD < 1: 47.1%
PLRDAK
SSD < 1: 46.6%
LRAKPD
SSD < 1: 46.4%
PKALDR
SSD < 1: 45.8%
DLRKPA
SSD < 1: 45.6%
LRAKDP
SSD < 1: 45.2%
RPALKD
SSD < 1: 45.1%
RKADPL
SSD < 1: 44.7%
ADPLRK
SSD < 1: 44.7%
KARDLP
SSD < 1: 44.7%
RPKDAL
SSD < 1: 44.2%
PADKLR
SSD < 1: 44.2%
RLKPDA
SSD < 1: 44.0%
RKDLAP
SSD < 1: 43.9%
DALPKR
SSD < 1: 43.9%
LRPKAD
SSD < 1: 43.7%
ALRPKD
SSD < 1: 43.2%
DRAKLP
SSD < 1: 42.9%
DPLKRA
SSD < 1: 42.7%
ALPKRD
SSD < 1: 42.6%
RPDKLA
SSD < 1: 42.5%
LKPRAD
SSD < 1: 42.4%
PRKADL
SSD < 1: 42.3%
DAPKLR
SSD < 1: 42.3%
PADLKR
SSD < 1: 42.2%
KLRPAD
SSD < 1: 42.1%
ALKPRD
SSD < 1: 42.1%
KAPLDR
SSD < 1: 41.9%
PRLAKD
SSD < 1: 41.8%
LPDKRA
SSD < 1: 41.4%
ALDRPK
SSD < 1: 41.4%
ALPDKR
SSD < 1: 40.9%
Distance [nm]
Time [ns]

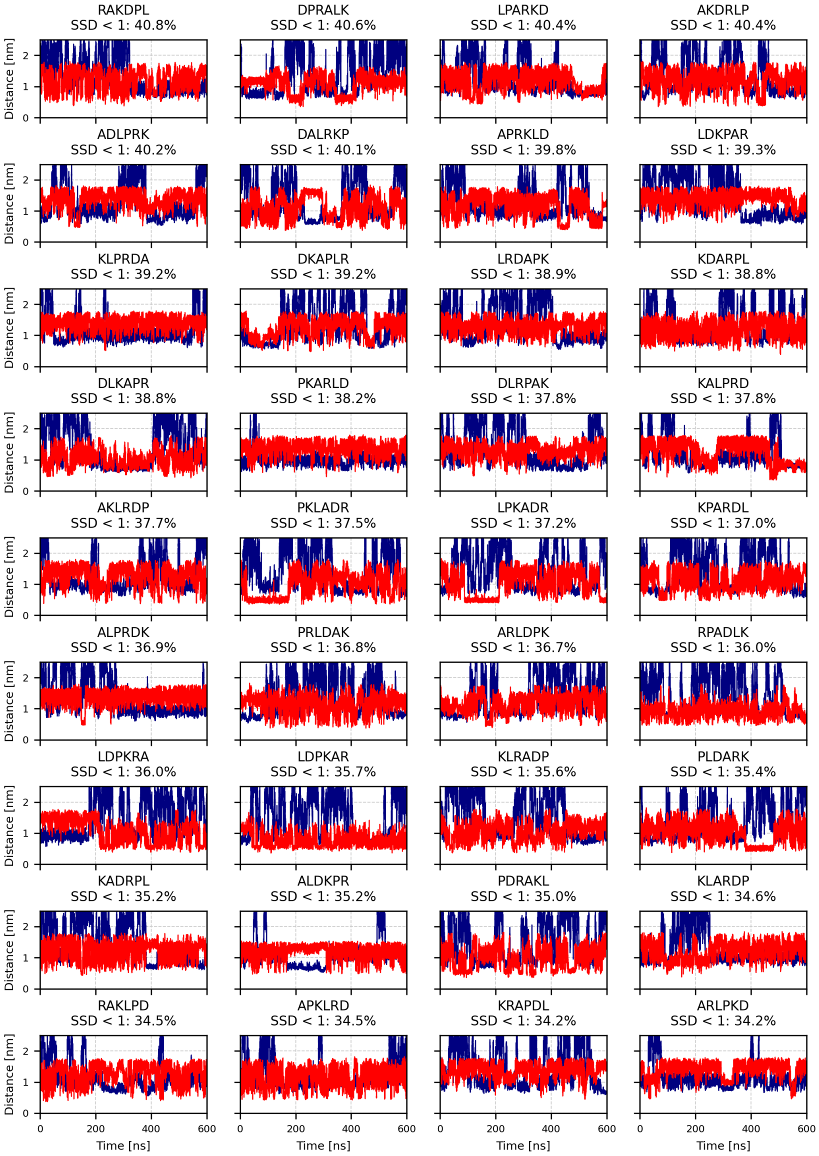
RAKDPL
SSD < 1: 40.8%
DPRALK
SSD < 1: 40.6%
LPARKD
SSD < 1: 40.4%
AKDRLP
SSD < 1: 40.4%
ADLPRK
SSD < 1: 40.2%
DALRKP
SSD < 1: 40.1%
APRKLD
SSD < 1: 39.8%
LDKPAR
SSD < 1: 39.3%
KLPRDA
SSD < 1: 39.2%
DKAPLR
SSD < 1: 39.2%
LRDAPK
SSD < 1: 38.9%
KDARPL
SSD < 1: 38.8%
DLKAPR
SSD < 1: 38.8%
PKARLD
SSD < 1: 38.2%
DLRPAK
SSD < 1: 37.8%
KALPRD
SSD < 1: 37.8%
AKLRDP
SSD < 1: 37.7%
PKLADR
SSD < 1: 37.5%
LPKADR
SSD < 1: 37.2%
KPARDL
SSD < 1: 37.0%
ALPRDK
SSD < 1: 36.9%
PRLDAK
SSD < 1: 36.8%
ARLDPK
SSD < 1: 36.7%
RPADLK
SSD < 1: 36.0%
LDPKRA
SSD < 1: 36.0%
LDPKAR
SSD < 1: 35.7%
KLRADP
SSD < 1: 35.6%
PLDARK
SSD < 1: 35.4%
KADRPL
SSD < 1: 35.2%
ALDKPR
SSD < 1: 35.2%
PDRAKL
SSD < 1: 35.0%
KLARDP
SSD < 1: 34.6%
RAKLPD
SSD < 1: 34.5%
APKLRD
SSD < 1: 34.5%
KRAPDL
SSD < 1: 34.2%
ARLPKD
SSD < 1: 34.2%
Distance [nm]
Time [ns]

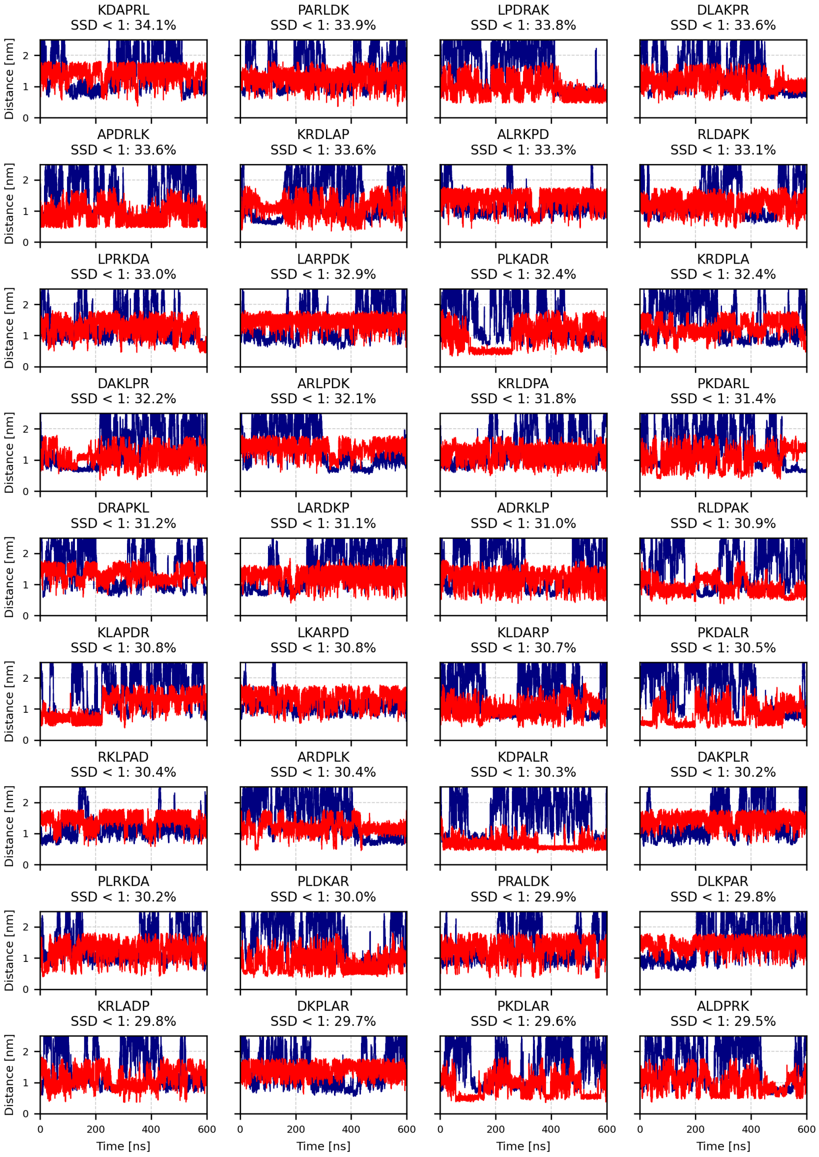
KDAPRL
SSD < 1: 34.1%
PARLDK
SSD < 1: 33.9%
LPDRAK
SSD < 1: 33.8%
DLAKPR
SSD < 1: 33.6%
APDRLK
SSD < 1: 33.6%
KRDLAP
SSD < 1: 33.6%
ALRKPD
SSD < 1: 33.3%
RLDAPK
SSD < 1: 33.1%
LPRKDA
SSD < 1: 33.0%
LARPDK
SSD < 1: 32.9%
PLKADR
SSD < 1: 32.4%
KRDPLA
SSD < 1: 32.4%
DAKLPR
SSD < 1: 32.2%
ARLPDK
SSD < 1: 32.1%
KRLDPA
SSD < 1: 31.8%
PKDARL
SSD < 1: 31.4%
DRAPKL
SSD < 1: 31.2%
LARDKP
SSD < 1: 31.1%
ADRKLP
SSD < 1: 31.0%
RLDPAK
SSD < 1: 30.9%
KLAPDR
SSD < 1: 30.8%
LKARPD
SSD < 1: 30.8%
KLDARP
SSD < 1: 30.7%
PKDALR
SSD < 1: 30.5%
RKLPAD
SSD < 1: 30.4%
ARDPLK
SSD < 1: 30.4%
KDPALR
SSD < 1: 30.3%
DAKPLR
SSD < 1: 30.2%
PLRKDA
SSD < 1: 30.2%
PLDKAR
SSD < 1: 30.0%
PRALDK
SSD < 1: 29.9%
DLKPAR
SSD < 1: 29.8%
KRLADP
SSD < 1: 29.8%
DKPLAR
SSD < 1: 29.7%
PKDLAR
SSD < 1: 29.6%
ALDPRK
SSD < 1: 29.5%
Distance [nm]
Time [ns]
0
1
2
200
400
600

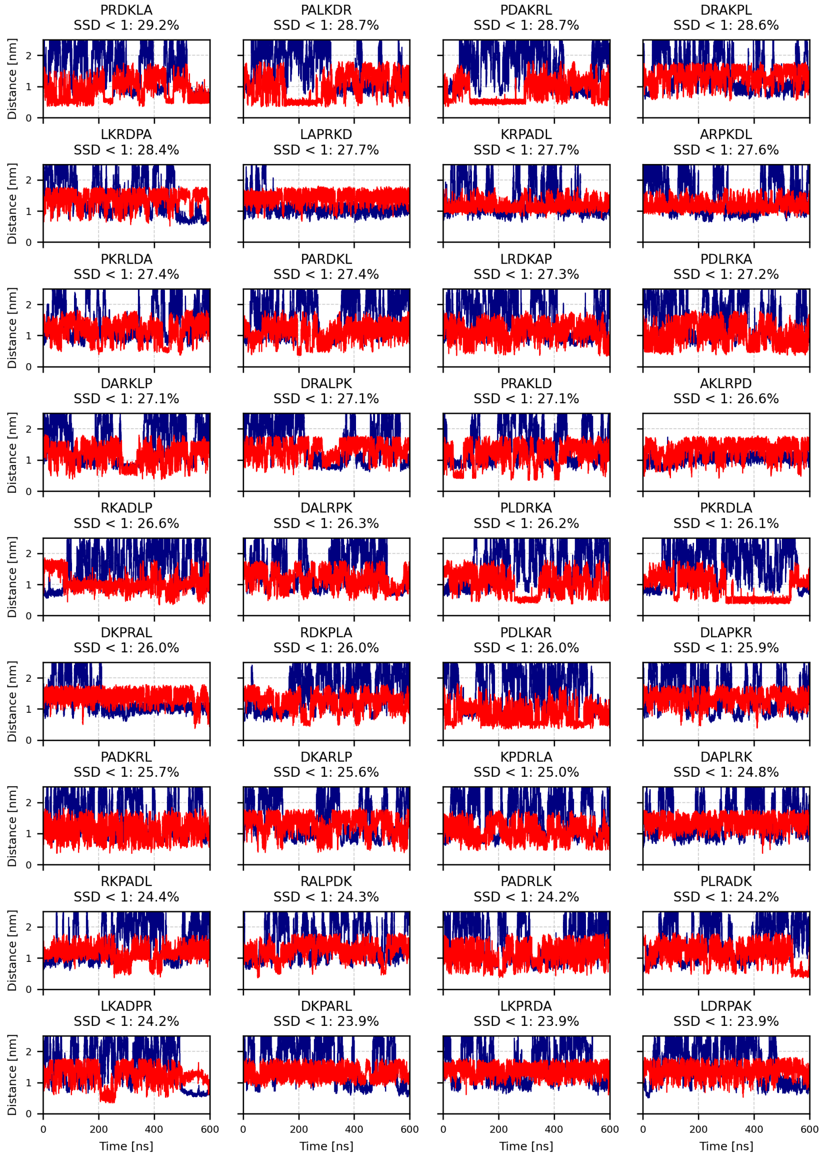
PRDKLA
SSD < 1: 29.2%
PALKDR
SSD < 1: 28.7%
PDAKRL
SSD < 1: 28.7%
DRAKPL
SSD < 1: 28.6%
LKRDPA
SSD < 1: 28.4%
LAPRKD
SSD < 1: 27.7%
KRPADL
SSD < 1: 27.7%
ARPKDL
SSD < 1: 27.6%
PKRLDA
SSD < 1: 27.4%
PARDKL
SSD < 1: 27.4%
LRDKAP
SSD < 1: 27.3%
PDLRKA
SSD < 1: 27.2%
DARKLP
SSD < 1: 27.1%
DRALPK
SSD < 1: 27.1%
PRAKLD
SSD < 1: 27.1%
AKLRPD
SSD < 1: 26.6%
RKADLP
SSD < 1: 26.6%
DALRPK
SSD < 1: 26.3%
PLDRKA
SSD < 1: 26.2%
PKRDLA
SSD < 1: 26.1%
DKPRAL
SSD < 1: 26.0%
RDKPLA
SSD < 1: 26.0%
PDLKAR
SSD < 1: 26.0%
DLAPKR
SSD < 1: 25.9%
PADKRL
SSD < 1: 25.7%
DKARLP
SSD < 1: 25.6%
KPDRLA
SSD < 1: 25.0%
DAPLRK
SSD < 1: 24.8%
RKPADL
SSD < 1: 24.4%
RALPDK
SSD < 1: 24.3%
PADRLK
SSD < 1: 24.2%
PLRADK
SSD < 1: 24.2%
LKADPR
SSD < 1: 24.2%
DKPARL
SSD < 1: 23.9%
LKPRDA
SSD < 1: 23.9%
LDRPAK
SSD < 1: 23.9%
Distance [nm]
Time [ns]
0
1
2
200
400
600

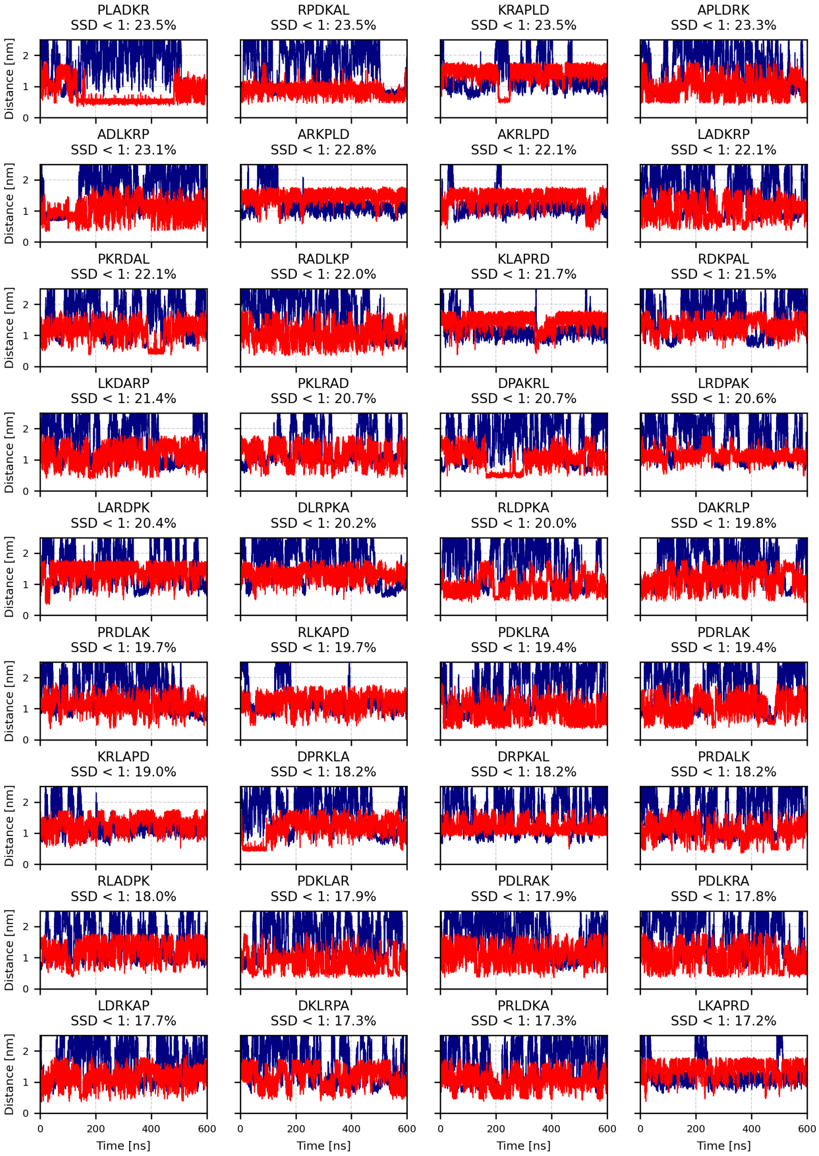
PLADKR
SSD < 1: 23.5%
RPDKAL
SSD < 1: 23.5%
KRAPLD
SSD < 1: 23.5%
APLDRK
SSD < 1: 23.3%
ADLKRP
SSD < 1: 23.1%
ARKPLD
SSD < 1: 22.8%
AKRLPD
SSD < 1: 22.1%
LADKRP
SSD < 1: 22.1%
PKRDAL
SSD < 1: 22.1%
RADLKP
SSD < 1: 22.0%
KLAPRD
SSD < 1: 21.7%
RDKPAL
SSD < 1: 21.5%
LKDARP
SSD < 1: 21.4%
PKLRAD
SSD < 1: 20.7%
DPAKRL
SSD < 1: 20.7%
LRDPAK
SSD < 1: 20.6%
LARDPK
SSD < 1: 20.4%
DLRPKA
SSD < 1: 20.2%
RLDPKA
SSD < 1: 20.0%
DAKRLP
SSD < 1: 19.8%
PRDLAK
SSD < 1: 19.7%
RLKAPD
SSD < 1: 19.7%
PDKLRA
SSD < 1: 19.4%
PDRLAK
SSD < 1: 19.4%
KRLAPD
SSD < 1: 19.0%
DPRKLA
SSD < 1: 18.2%
DRPKAL
SSD < 1: 18.2%
PRDALK
SSD < 1: 18.2%
RLADPK
SSD < 1: 18.0%
PDKLAR
SSD < 1: 17.9%
PDLRAK
SSD < 1: 17.9%
PDLKRA
SSD < 1: 17.8%
LDRKAP
SSD < 1: 17.7%
DKLRPA
SSD < 1: 17.3%
PRLDKA
SSD < 1: 17.3%
LKAPRD
SSD < 1: 17.2%
Distance [nm]
Time [ns]
0
200
400
600
0
1
2

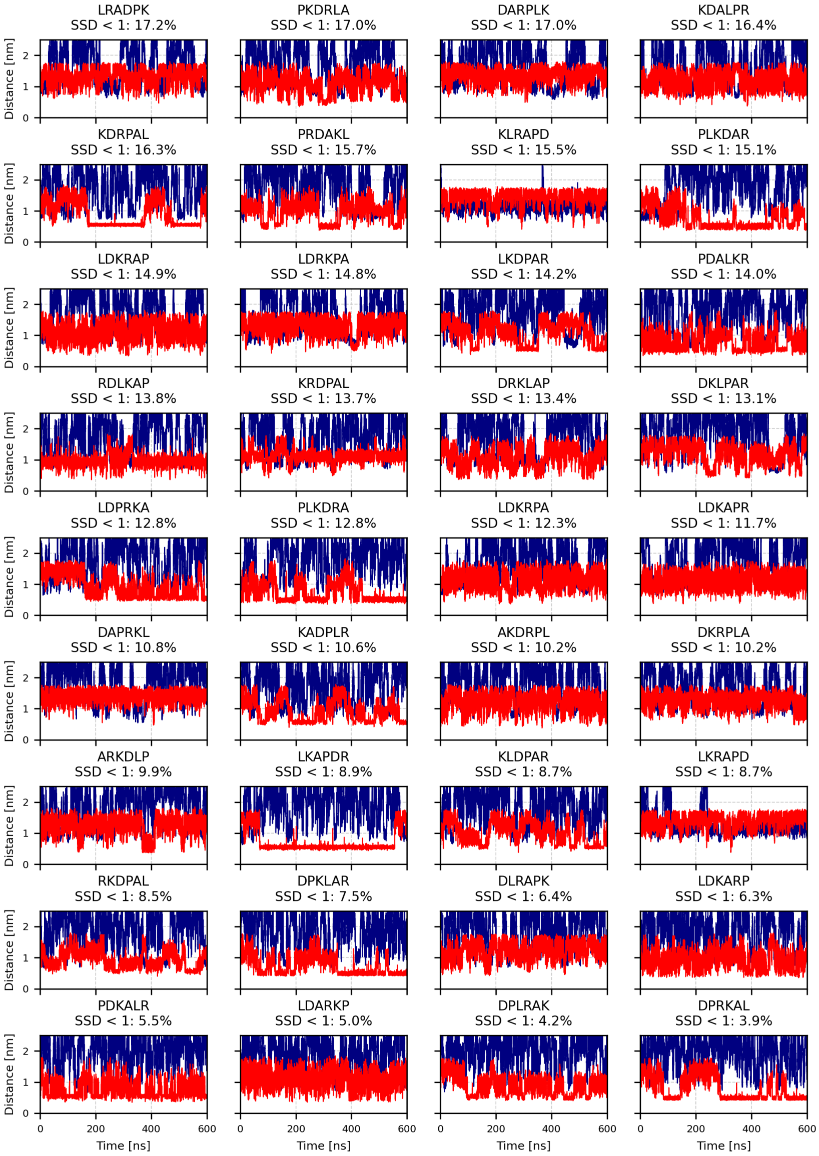

Fig. S1 SSD and EED for all 360 sequences in this study.

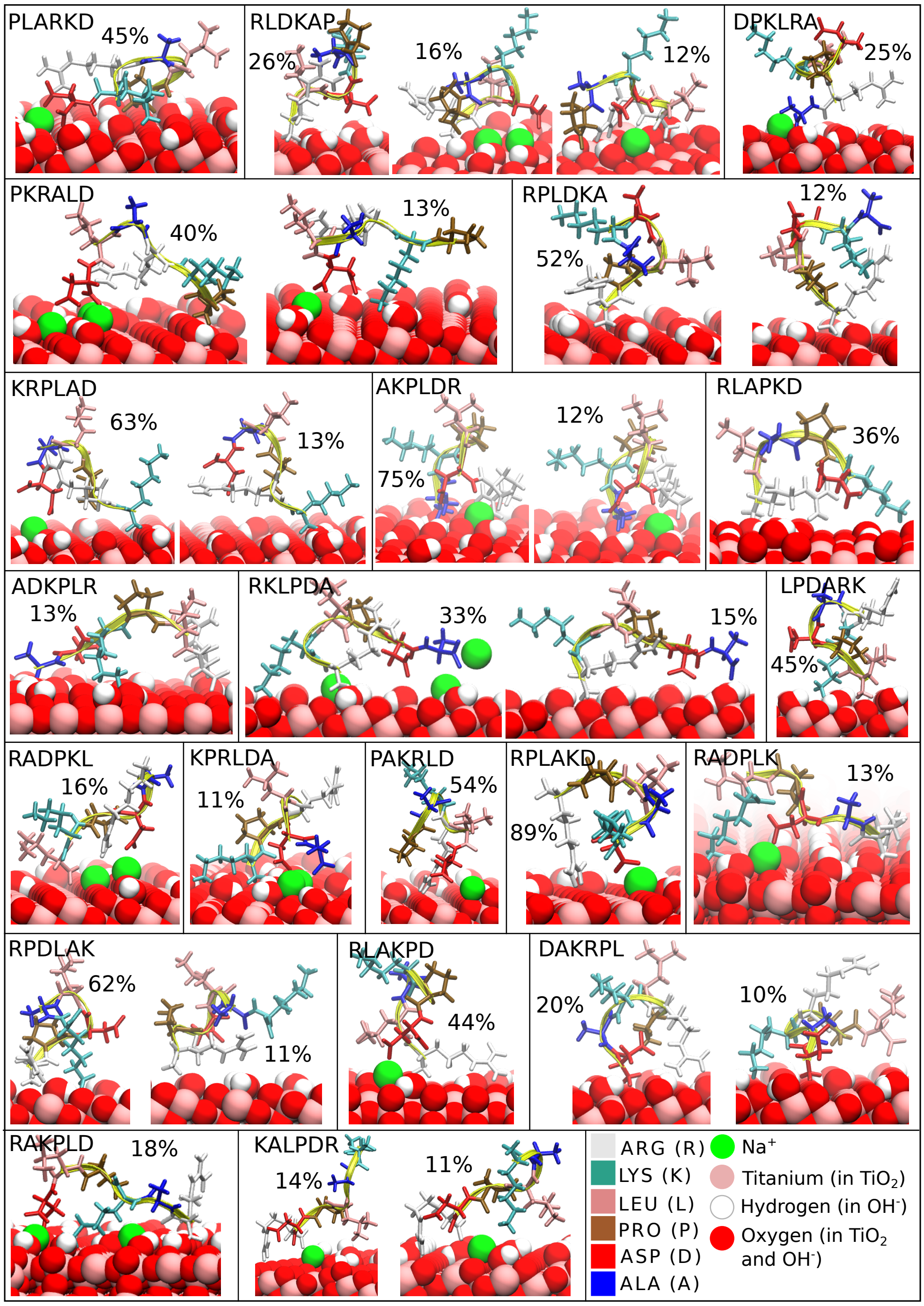

Fig. S2 Bound-state conformations for the sequences with SSD < 1 nm for more than 90% of the simulation time (first 24 sequences in Table 2) obtained from clustering. Only the most populated clusters (more than 10%) for each sequence is shown together with their population. The TiO_2 surface atoms—including titanium, oxygen, and the oxygen and hydrogen atoms of surface hydroxyl groups—as well as sodium ions (Na^+), are depicted in ball representation and marked with circle shapes, following a consistent color scheme. All the amino acid residues displayed in cylinder representation, color-coded and indicated with square shapes.

Sequence	% bound	RDF	Sequence	% bound	RDF	Sequence	% bound	RDF
PLARKD	100.00	K_OF_NZ	LRPKDA	73.60	L_OF_N	KALRPD	56.09	K_OF_N
RLDKAP	99.56	R_OF_N	AKLDRP	72.67	A_OF_N	APLRDK	55.88	A_OF_N
DPKLRA	99.14	A_OF_OC2	APLRKD	72.45	A_OF_N	KPLDAR	55.79	K_OF_NZ
PKRALD	98.36	P_OF_N	KADPRL	72.44	K_OF_N	LAKDRP	55.58	L_OF_N
RPLDKA	98.09	R_OF_N	LADRPK	72.07	L_OF_N	RPKLAD	54.70	K_OF_NZ
PRLKAD	97.90	D_OF_OC2	KADLPR	71.84	K_OF_N	ARDLPK	54.54	A_OF_N
KRPLAD	97.76	K_OF_N	KPALDR	71.77	K_OF_NZ	LRKDPA	54.49	K_OF_NZ
AKPLDR	97.52	A_OF_N	RPDALK	71.68	R_OF_N	KPLRAD	54.39	K_OF_N
RLAPKD	96.76	R_OF_N	LKPDRA	71.49	L_OF_N	RDPLKA	54.35	R_OF_N
ADKPLR	96.70	R_OB_OC2	LPAKDR	70.92	K_OF_NZ	LKPADR	54.23	L_OF_N
RKLPDA	96.51	R_OF_N	RPKADL	70.23	R_OF_N	LPKDRA	54.05	L_OF_N
LPDARK	96.41	L_OF_N	APKLDR	70.07	A_OF_N	ARPLDK	53.04	A_OF_N
RADPKL	96.10	L_OF_OC1	APDLKR	68.93	A_OF_N	KDRLAP	52.38	K_OF_N
KPRLDA	95.93	K_OF_N	KPRALD	67.92	K_OF_NZ	AKDPRL	52.03	A_OF_N
PAKRLD	95.63	P_OF_N	LRPAKD	67.82	L_OF_N	RAPLDK	52.03	R_OF_N
RPLAKD	95.55	R_OB_NH2	DARKPL	67.20	D_OF_N	RKPLDA	51.52	R_OF_N
RADPLK	94.34	R_OF_N	KDAPLR	67.09	K_OF_N	LPADKR	51.07	L_OF_N
RPDLAK	93.99	R_OF_N	PKADLR	67.03	P_OF_N	DRLPKA	50.80	D_OF_N
RLAKPD	93.02	R_OF_N	KRALPD	66.94	K_OF_N	KLPARD	49.92	K_OF_N
DAKRPL	92.74	D_OF_N	RKALPD	66.86	R_OF_N	KAPLRD	49.72	K_OF_N
RAKPLD	92.64	R_OF_N	LRDAKP	66.32	L_OF_N	KRPALD	49.69	D_OF_OC1
RKAPDL	91.96	R_OF_N	DLPRKA	65.59	D_OF_N	LRDPKA	49.29	L_OF_N
KALPDR	91.73	R_OB_NH2	AKRDPL	65.20	A_OF_N	KRPDAL	48.51	K_OF_N
PDKRAL	91.40	P_OF_N	KPALRD	65.15	K_OF_NZ	PRLADK	48.18	K_OF_NZ
RADKPL	88.43	R_OF_N	RKPDLA	64.58	R_OF_N	DRKLPA	48.11	D_OF_N
RPKLDA	87.53	R_OF_N	KLDPRA	64.35	K_OF_N	LRKPAD	47.85	K_OF_NZ
PDKRLA	87.53	P_OF_N	LPKRDA	64.08	L_OF_N	LRKPDA	47.84	K_OF_NZ
KRALDP	87.47	K_OF_N	KDRALP	63.99	K_OF_N	ARLDKP	47.54	A_OF_N
KLRPDA	87.30	K_OF_N	PRALKD	63.85	D_OF_OC1	LRDKPA	47.11	L_OF_N
APDKLR	87.26	A_OF_N	ADPRKL	63.66	A_OF_N	PLRDAK	46.56	P_OF_N
DPALKR	87.04	D_OF_N	RDKLPA	63.58	R_OF_N	LRAKPD	46.39	K_OF_NZ
RKALDP	86.32	K_OF_NZ	KPLRDA	63.05	D_OF_OD2	PKALDR	45.83	P_OF_N
RKAPLD	86.03	R_OF_N	KRLDAP	62.81	K_OF_N	DLRKPA	45.60	D_OF_N
KPDLAR	85.32	K_OF_NZ	DLPRAK	62.68	D_OF_N	LRAKDP	45.20	L_OF_N
RPALDK	83.82	R_OF_N	RPADKL	62.18	R_OF_N	RPALKD	45.14	D_OF_OD1
LPAKRD	83.22	L_OF_N	PARLKD	62.01	P_OF_N	RKADPL	44.71	R_OF_N
AKLPRD	83.08	A_OF_N	ALKPDR	61.99	A_OF_N	ADPLRK	44.70	K_OF_NZ
LKRDAP	83.05	K_OF_NZ	ARPDKL	61.98	A_OF_N	KARDLP	44.69	K_OF_N
PKLDRA	81.84	P_OF_N	ALDKRP	61.96	A_OF_N	RPKDAL	44.20	K_OF_NZ
KLPADR	81.09	K_OF_N	KDLRPA	61.56	K_OF_N	PADKLR	44.16	K_OF_NZ
LDRPKA	80.73	L_OF_N	LPADRK	60.86	D_OF_OD1	RLKPDA	44.02	K_OF_NZ
RPLKDA	80.72	R_OF_N	ALKRDP	60.86	D_OF_OD1	RKDLAP	43.93	R_OF_N
KDRAPL	80.71	K_OF_N	DALKRP	60.65	D_OF_N	DALPKR	43.85	D_OF_N
LAKRDP	80.69	L_OF_N	ALRPDK	60.64	A_OF_N	LRPKAD	43.71	D_OF_OD1
KARPDL	80.49	K_OF_N	DPKRAL	60.29	D_OF_N	ALRPKD	43.20	D_OF_OD2
KLDRPA	80.47	K_OF_N	DKARPL	60.00	D_OF_N	DRAKLP	42.86	D_OF_N
APRDKL	79.95	K_OF_NZ	PKLARD	59.95	D_OF_OC1	DPLKRA	42.71	D_OF_N
DRKALP	79.90	D_OF_N	PALRKD	59.84	P_OF_N	ALPKRD	42.56	K_OF_NZ
ADRLKP	79.79	A_OF_N	LADPKR	59.82	L_OF_N	RPDKLA	42.45	R_OF_N
AKDPLR	78.40	A_OF_N	RPDLKA	59.75	K_OF_NZ	LKPRAD	42.39	D_OF_OD2
DRLKAP	77.49	D_OF_N	PARKLD	59.46	P_OF_N	PRKADL	42.34	P_OF_N
APLDKR	76.84	K_OF_NZ	KDPARL	59.42	K_OF_N	DAPKLR	42.30	D_OF_N
ARPKLD	76.70	A_OF_N	DLAKRP	58.66	D_OF_N	PADLKR	42.19	K_OF_NZ
DRKPAL	76.63	D_OF_N	PDARKL	58.41	P_OF_N	KLRPAD	42.15	K_OF_NZ
KLDRAP	76.26	K_OF_N	KPRADL	58.20	K_OF_NZ	ALKPRD	42.09	D_OF_OC2
KRDALP	76.14	K_OF_NZ	RLDKPA	57.10	R_OF_N	KAPLDR	41.94	R_OF_OC1
DRLAKP	75.37	D_OF_N	RDPKAL	56.75	R_OF_N	PRLAKD	41.79	D_OF_OC2
PKARDL	74.02	P_OF_N	ARPLKD	56.73	A_OF_N	LPDKRA	41.38	K_OF_NZ
PDAKLR	73.94	P_OF_N	RDALPK	56.55	R_OF_N	ALDRPK	41.38	A_OF_N
ADKLRP	73.64	A_OF_N	RDKALP	56.32	R_OF_N	ALPDKR	40.86	A_OF_N

Sequence	% bound	RDF	Sequence	% bound	RDF	Sequence	% bound	RDF
RAKDPL	40.77	R_OF_N	RKLPAD	30.45	D_OF_OD2	LKDARP	21.45	K_OF_NZ
DPRALK	40.60	D_OF_N	ARDPLK	30.44	A_OF_N	PKLRAD	20.74	D_OF_OC2
LPARKD	40.44	L_OF_N	KDPALR	30.27	K_OF_NZ	DPAKRL	20.69	K_OF_NZ
AKDRLP	40.35	A_OF_N	DAKPLR	30.18	D_OF_N	LRDPAK	20.63	K_OF_NZ
ADLPRK	40.22	A_OF_N	PLRKDA	30.16	D_OF_OD2	LARDPK	20.45	L_OF_N
DALRKP	40.14	D_OF_N	PLDKAR	30.04	R_OF_OC1	DLRPKA	20.25	D_OF_N
APRKLD	39.84	K_OF_NZ	PRALDK	29.86	K_OF_NZ	RLDPKA	19.97	K_OF_NZ
LDKPAR	39.34	L_OF_N	DLKPAR	29.82	D_OF_N	DAKRLP	19.82	K_OF_NZ
KLPRDA	39.22	K_OF_N	KRLADP	29.76	P_OF_OC1	PRDLAK	19.73	K_OF_NZ
DKAPLR	39.18	D_OF_N	DKPLAR	29.71	D_OF_N	RLKAPD	19.73	D_OF_OD1
LRDAPK	38.86	L_OF_N	PKDLAR	29.64	R_OF_OC1	PDKLRA	19.42	P_OF_N
KDARPL	38.81	K_OF_N	ALDPRK	29.49	A_OF_N	PDRLAK	19.36	P_OF_N
DLKAPR	38.79	D_OF_N	PRDKLA	29.16	K_OF_NZ	KRLAPD	19.03	D_OF_OD2
PKARLD	38.18	R_OF_NH1	PALKDR	28.66	R_OF_OC2	DPRKLA	18.23	D_OF_N
DLRPAK	37.79	D_OF_N	PDAKRL	28.66	P_OF_N	DRPKAL	18.23	K_OF_NZ
KALPRD	37.77	K_OF_NZ	DRAKPL	28.61	D_OF_N	PRDALK	18.22	K_OF_NZ
AKLRDP	37.66	A_OF_N	LKRDPA	28.45	L_OF_N	RLADPK	17.99	K_OF_NZ
PKLADR	37.48	P_OF_N	LAPRKD	27.72	D_OF_OD2	PDKLAR	17.95	K_OF_NZ
LPKADR	37.25	K_OF_NZ	KRPADL	27.68	L_OF_OC2	PDLRAK	17.88	P_OF_N
KPARDL	37.00	K_OF_NZ	ARPKDL	27.56	A_OF_N	PDLKRA	17.80	K_OF_NZ
ALPRDK	36.89	K_OF_NZ	PKRLDA	27.44	A_OF_OC2	LDRKAP	17.72	K_OF_NZ
PRLDAK	36.84	K_OF_NZ	PARDKL	27.40	K_OF_NZ	DKLRPA	17.32	K_OF_NZ
ARLDPK	36.66	A_OF_N	LRDKAP	27.27	K_OF_NZ	PRLDKA	17.29	K_OF_NZ
RPADLK	36.05	R_OF_N	PDLRKA	27.21	K_OF_NZ	LKAPRD	17.19	D_OF_OD
LDPKRA	35.98	L_OF_N	DARKLP	27.12	D_OF_N	LRADPK	17.18	K_OF_NZ
LDPKAR	35.72	K_OF_NZ	DRALPK	27.08	D_OF_N	PKDRLA	16.98	P_OF_N
KLRADP	35.59	K_OF_NZ	PRAKLD	27.05	K_OF_NZ	DARPLK	16.97	K_OF_NZ
PLDARK	35.39	K_OF_NZ	AKLRPD	26.62	D_OF_OD1	KDALPR	16.45	R_OF_OC
KADRPL	35.21	K_OF_N	RKADLP	26.55	K_OF_NZ	KDRPAL	16.30	K_OF_NZ
ALDKPR	35.16	A_OF_N	DALRPK	26.31	D_OF_N	PRDAKL	15.74	K_OF_NZ
PDRAKL	35.00	K_OF_NZ	PLDRKA	26.24	P_OF_N	KLRAPD	15.46	D_OF_OC
KLARDP	34.64	P_OF_OC2	PKRDLA	26.15	R_OF_NH2	PLKDAR	15.06	K_OF_NZ
RAKLPD	34.49	K_OF_NZ	DKPRAL	26.04	D_OF_N	LDKRAP	14.87	K_OF_NZ
APKLRD	34.47	D_OF_OC2	RDKPLA	26.02	R_OF_N	LDRKPA	14.84	L_OF_N
KRAPDL	34.25	K_OF_N	PDLKAR	25.96	K_OF_NZ	LKDPAR	14.20	L_OF_N
ARLPKD	34.24	D_OF_OC2	DLAPKR	25.90	K_OF_NZ	PDALKR	14.02	K_OF_NZ
KDAPRL	34.09	K_OF_N	PADKRL	25.74	P_OF_N	RDLKAP	13.83	K_OF_NZ
PARLDK	33.87	R_OF_NH1	DKARLP	25.61	D_OF_N	KRDPAL	13.68	K_OF_NZ
LPDRAK	33.83	K_OF_NZ	KPDRLA	25.04	K_OF_NZ	DRKLAP	13.41	K_OF_NZ
DLAKPR	33.61	D_OF_N	DAPLRK	24.76	D_OF_N	DKLPAR	13.05	D_OF_N
APDRLK	33.58	K_OF_NZ	RKPADL	24.37	K_OF_NZ	LDPRKA	12.81	A_OF_OC1
KRDLAP	33.57	K_OF_N	RALPDK	24.33	K_OF_NZ	PLKDRA	12.80	K_OF_NZ
ALRKPD	33.31	K_OF_NZ	PADRLK	24.19	K_OF_NZ	LDKRPA	12.30	A_OF_OC1
RLDAPK	33.15	R_OF_N	PLRADK	24.17	K_OF_NZ	LDKAPR	11.70	K_OF_NZ
LPRKDA	32.95	K_OF_NZ	LKADPR	24.17	L_OF_N	DAPRKL	10.84	D_OF_N
LARPDK	32.94	L_OF_N	DKPARL	23.90	K_OF_NZ	KADPLR	10.60	K_OF_NZ
PLKADR	32.43	R_OF_OC2	LKPRDA	23.90	K_OF_NZ	AKDRPL	10.25	L_OF_OC2
KRDPLA	32.39	K_OF_NZ	LDRPAK	23.86	L_OF_N	DKRPLA	10.24	K_OF_NZ
DAKLPR	32.22	D_OF_N	PLADKR	23.54	K_OF_NZ	ARKDLP	9.90	A_OF_N
ARLPDK	32.14	A_OF_N	RPDKAL	23.54	R_OB_NH2	LKAPDR	8.90	D_OF_OD1
KRLDPA	31.84	K_OF_N	KRAPLD	23.49	K_OF_NZ	KLDPAR	8.72	K_OF_NZ
PKDARL	31.45	P_OF_N	APLDRK	23.31	A_OF_N	LKRAPD	8.65	D_OF_OD2
DRAPKL	31.20	D_OF_N	ADLKRP	23.11	A_OF_N	RKDPAL	8.46	R_OF_N
LARDKP	31.05	L_OF_N	ARKPLD	22.82	D_OF_OD2	DPKLAR	7.53	K_OF_NZ
ADRKLP	30.99	A_OF_N	AKRLPD	22.13	D_OF_OC2	DLRAPK	6.42	K_OF_NZ
RLDPAK	30.90	R_OF_N	LADKRP	22.10	L_OF_N	LDKARP	6.26	K_OF_NZ
KLAPDR	30.81	K_OF_N	PKRDAL	22.05	P_OF_N	PDKALR	5.48	R_OF_OC2
LKARPD	30.75	D_OF_OC2	RADLKP	21.98	R_OF_N	LDARKP	5.02	P_OF_OC2
KLDARP	30.73	K_OF_N	KLAPRD	21.72	D_OF_OC1	DPLRAK	4.25	K_OF_NZ
PKDALR	30.54	P_OF_N	RDKPAL	21.47	K_OF_NZ	DPRKAL	3.89	R_OF_NH1

Table S1 Peptides binding ranking according to the percentage of time to have SSD value less than 1 nm over simulation time, and notation for RDFs having highest maximum.

Sequence	EED_a	std_a	EED_d	std_d	Sequence	EED_a	std_a	EED_d	std_d	Sequence	EED_a	std_a	EED_d	std_d
PLARKD	1.16	0.22	1.15	-	APRDKL	0.79	0.23	1.09	0.32	KRLDAP	1.03	0.23	1.00	0.24
RLDKAP	0.98	0.21	1.30	0.25	DRKALP	1.26	0.23	1.30	0.29	DLPRAK	1.36	0.19	1.44	0.14
DPKLRA	0.94	0.17	1.49	0.15	ADRLKP	1.01	0.24	1.19	0.23	RPADKL	0.81	0.24	0.82	0.22
PKRALD	1.15	0.11	1.32	0.18	AKDPLR	1.11	0.30	1.24	0.31	PARLKD	1.16	0.17	1.47	0.18
RPLDKA	0.79	0.20	1.20	0.21	DRLKAP	1.16	0.27	1.38	0.18	ALKPDR	1.46	0.16	1.40	0.17
PRLKAD	1.31	0.26	1.46	0.13	APLDKR	0.70	0.14	0.77	0.26	ARPDKL	1.12	0.18	1.10	0.12
KRPLAD	1.05	0.07	1.23	0.17	ARPKLD	1.01	0.28	1.18	0.28	ALDKRP	1.12	0.21	1.07	0.31
AKPLDR	0.68	0.15	1.46	0.13	DRKPAL	1.39	0.17	1.52	0.10	KDLRPA	1.15	0.26	1.25	0.26
RLAPKD	1.17	0.21	1.46	0.10	KLDRAP	0.99	0.18	1.16	0.26	LPADRK	0.89	0.18	0.92	0.28
ADKPLR	1.39	0.17	1.52	0.15	KRDALP	1.05	0.16	0.90	0.24	ALKRDP	1.30	0.26	1.30	0.26
RKLPDA	1.17	0.16	1.48	0.14	DRLAKP	1.11	0.26	1.21	0.29	DALKRP	1.20	0.20	1.19	0.29
LPDARK	0.84	0.23	1.34	0.16	PKARDL	1.30	0.23	1.34	0.22	ALRPDK	1.30	0.17	1.39	0.16
RADPKL	1.26	0.22	1.52	0.10	PDAKLR	0.82	0.23	0.95	0.29	DPKRAL	1.25	0.23	1.32	0.21
KPRLDA	1.04	0.26	1.33	0.15	ADKLRP	0.90	0.22	1.05	0.28	DKARPL	1.20	0.24	1.21	0.32
PAKRLD	0.84	0.39	1.35	0.20	LRPKDA	1.17	0.15	1.25	0.19	PKLARD	0.82	0.35	0.68	0.35
RPLAKD	0.66	0.10	0.92	0.27	AKLDRP	1.20	0.24	1.26	0.27	PALRKD	0.95	0.34	1.27	0.33
RADPLK	1.30	0.22	1.43	0.14	APLRKD	0.98	0.11	1.29	0.20	LADPKR	1.09	0.20	1.13	0.33
RPDLAK	0.76	0.13	0.82	0.15	KADPRL	1.14	0.30	1.20	0.28	RPDLKA	0.76	0.21	0.87	0.25
RLAKPD	0.96	0.17	1.16	0.30	LADRPK	1.38	0.27	1.29	0.23	PARKLD	1.26	0.32	1.32	0.34
DAKRPL	1.15	0.17	1.40	0.17	KADLPR	1.29	0.23	1.34	0.23	KDPARL	0.86	0.20	0.94	0.35
RAKPLD	1.22	0.19	1.46	0.10	KPALDR	0.71	0.14	0.82	0.22	DLAKRP	0.90	0.25	1.14	0.29
RKAPDL	1.31	0.27	1.46	0.14	RPDALK	0.86	0.15	0.90	0.16	PDARKL	0.85	0.27	0.99	0.34
KALPDR	1.33	0.19	1.44	0.13	LKPDRA	1.14	0.17	1.19	0.21	KPRADL	0.78	0.21	1.05	0.33
PDKRAL	1.24	0.27	1.31	0.22	LPAKDR	0.77	0.21	0.98	0.31	RLDKPA	1.29	0.23	1.15	0.27
RADKPL	1.11	0.26	1.36	0.19	RPKADL	0.72	0.10	0.77	0.13	RDPKAL	0.96	0.18	0.98	0.28
RPKLDA	0.74	0.16	0.74	0.20	APKLDR	0.93	0.26	1.13	0.30	ARPLKD	1.12	0.14	1.27	0.20
PDKRLA	1.21	0.25	1.39	0.15	APDLKR	0.83	0.22	0.84	0.28	RDALPK	1.07	0.20	1.17	0.25
KRALDP	1.37	0.10	1.09	0.22	KPRALD	1.12	0.40	1.35	0.29	RDKALP	0.87	0.12	0.92	0.11
KLRPDA	1.15	0.19	1.26	0.19	LRPAKD	1.13	0.12	1.22	0.19	KALRPD	1.09	0.22	1.39	0.23
APDKLR	0.87	0.21	1.07	0.23	DARKPL	1.20	0.12	1.25	0.25	APLRDK	1.02	0.25	1.27	0.18
DPALKR	0.92	0.18	1.21	0.23	KDAPLR	1.31	0.23	1.50	0.12	KPLDAR	0.72	0.15	0.76	0.17
RKALDP	0.96	0.36	1.14	0.32	PKADLR	1.09	0.25	0.94	0.43	LAKDRP	0.97	0.25	1.17	0.31
RKAPLD	1.00	0.16	1.34	0.21	KRALPD	1.22	0.16	1.33	0.24	RPKLAD	0.78	0.25	1.05	0.28
KPDLAR	0.69	0.11	0.73	0.15	RKALPD	1.07	0.32	1.30	0.29	ARDLPK	1.26	0.21	1.36	0.20
RPALDK	0.78	0.11	0.76	0.22	LRDAKP	1.28	0.16	1.23	0.26	LRKDPA	1.18	0.21	1.21	0.19
LPAKRD	1.21	0.19	1.24	0.24	DLPRKA	1.36	0.15	1.39	0.25	KPLRAD	0.86	0.28	1.18	0.26
AKLPRD	1.19	0.19	1.43	0.19	AKRDPL	1.29	0.21	1.46	0.18	RDPLKA	0.69	0.16	0.79	0.31
LKRDAP	1.56	0.10	1.32	0.17	KPALRD	1.02	0.30	1.32	0.29	LKPADR	1.18	0.17	1.32	0.18
PKLDRA	0.80	0.32	1.24	0.32	RKPDLA	1.12	0.16	1.14	0.17	LPKDRA	0.89	0.32	0.91	0.32
KLPADR	1.12	0.22	1.31	0.21	KLDPRA	1.15	0.33	1.06	0.33	ARPLDK	1.23	0.16	1.30	0.19
LDRPKA	1.30	0.21	1.46	0.13	LPKRDA	1.34	0.19	1.27	0.22	KDRLAP	0.94	0.17	1.04	0.22
RPLKDA	0.75	0.21	0.75	0.19	KDRALP	1.04	0.24	0.99	0.22	AKDPRL	0.81	0.25	1.16	0.25
KDRAPL	1.21	0.25	1.49	0.13	PRALKD	1.07	0.21	1.21	0.35	RAPLDK	1.00	0.26	1.13	0.30
LAKRDP	0.94	0.19	1.37	0.26	ADPRKL	1.01	0.24	1.01	0.32	RKPLDA	0.98	0.29	1.14	0.28
KARPDL	1.31	0.19	1.38	0.15	RDKLPA	1.09	0.24	1.09	0.31	LPADKR	0.79	0.23	0.70	0.16
KLDRPA	1.39	0.20	1.20	0.22	KPLRDA	0.94	0.29	1.07	0.33	DRLPKA	1.29	0.22	1.39	0.22

Table S2 Peptides binding ranking according to the percentage of time to have SSD value less than 1 nm over simulation time.
